# Interfacial Water Structure of Binary Liquid Mixtures Reflects Nonideal Behavior

**DOI:** 10.1021/acs.jpcb.1c06001

**Published:** 2021-09-10

**Authors:** Xiaoqing Yu, Takakazu Seki, Chun-Chieh Yu, Kai Zhong, Shumei Sun, Masanari Okuno, Ellen H. G. Backus, Johannes Hunger, Mischa Bonn, Yuki Nagata

**Affiliations:** † Max Planck Institute for Polymer Research, Ackermannweg 10, 55128 Mainz, Germany; ‡ University of Groningen, Zernike Institute for Advanced Materials, Nijenborgh 4, 9747 AG Groningen, The Netherlands; § Department of Physics, Applied Optics Beijing Area Major Laboratory, Beijing Normal University, 100875 Beijing, China; ∥ Department of Basic Science, Graduate School of Arts and Sciences, The University of Tokyo, Komaba, Meguro, 153-8902 Tokyo, Japan; ⊥ Department of Physical Chemistry, University of Vienna, Währinger Strasse 42, 1090 Vienna, Austria

## Abstract

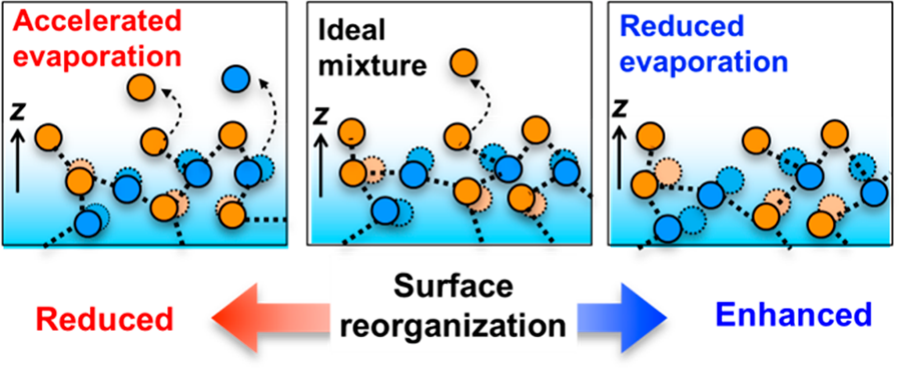

The evaporation of molecules from water–organic solute binary mixtures is key for both atmospheric and industrial processes such as aerosol formation and distillation. Deviations from ideal evaporation energetics can be assigned to intermolecular interactions in solution, yet evaporation occurs from the interface, and the poorly understood interfacial, rather than the bulk, structure of binary mixtures affects evaporation kinetics. Here we determine the interfacial structure of nonideal binary mixtures of water with methanol, ethanol, and formic acid, by combining surface-specific vibrational spectroscopy with molecular dynamics simulations. We find that the free, dangling OH groups at the interfaces of these differently behaving nonideal mixtures are essentially indistinguishable. In contrast, the ordering of hydrogen-bonded interfacial water molecules differs substantially at these three interfaces. Specifically, the interfacial water molecules become more disordered (ordered) in mixtures with methanol and ethanol (formic acid), showing higher (lower) vapor pressure than that predicted by Raoult’s law.

## I Introduction

Binary liquid mixtures typically deviate from ideal mixing in a thermodynamic context.^1,2^ In the bulk liquid phase, such nonideal interactions can be trivially understood, as they only require, in a mixture of A and B species, that the interactions between A and B differ from A–A and B–B interactions. A textbook example for nonideal mixtures where A–B interactions are stronger than A–A and B–B interactions in water–formic acid mixtures.^3^ As a result, the vapor pressure of water–formic acid mixtures is markedly lower than that predicted by Raoult’s law. Conversely, water–alcohol mixtures typically show excess vapor pressures.^4^ These excess interactions impact the energetics of the evaporation, which is directly relevant to distillation and purification processes in industrial processes and also to atmospheric processes.^5−11^

**Figure 1:**
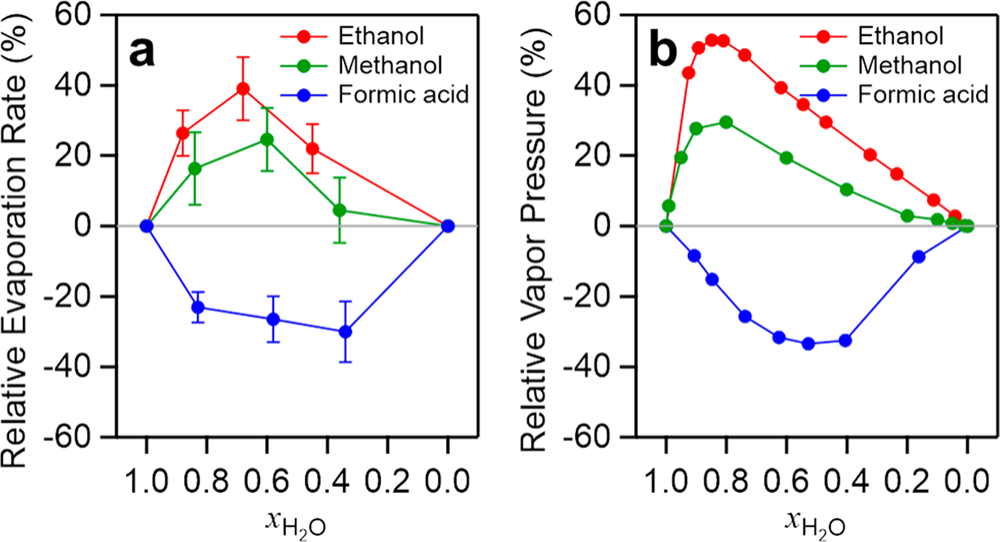
(a) Relative evaporation rate obtained from aqueous binary mixtures of methanol, ethanol, and formic acid vs water mole fraction, *x*_H_2_O_. The relative evaporation rate was calculated via Δ*k*(*x*_H_2_O)_ = *k*_H_2_O–org_(*x*_H_2_O_)/*k*_pred_(*x*_H_2_O_) – 1, where *k*_H_2_O–org_(*x*_H_2_O_) is the measured evaporation rate of the binary mixture and *k*_pred_(*x*_H_2_O_) is the predicted evaporation rate from the evaporation rate of pure water (*k*_H_2_O_) and pure organic species (*k*_org_): *k*_pred_(*x*_H_2_O_) = *x*_H_2_O_*k*_H_2_O_ + (1 – *x*_H_2_O_)*k*_org_. The error bar indicates the 95% confidence interval of the measured evaporation rate. (b) Relative vapor pressure for the binary mixtures at 333 K. The data were calculated via Δ*p*(*x*_H_2_O_) = *p*_H_2_O–org_(*x*_H_2_O_)/*p*_pred_(*x*_H_2_O_) – 1, where *p*_H_2_O–org_(*x*_H_2_O_) is real vapor pressure^3,4,29^ and *p*_pred_(*x*_H_2_O_) is the predicted one from Raoult’s law. See the Supporting Information for more details.

While these energetics have been traced to the intermolecular interactions in the bulk, evaporation takes place at the interface and molecular interactions^12^ and the abundancy of the molecules at the interface are decisive factors determining evaporation rates. The activation barrier for the evaporation process and thus evaporation rate of an ideal A–B liquid binary mixture vary linearly with the concentration of A and B species, when A–A, A–B, and B–B interactions are identical, as defined in Raoult’s law. However, also the evaporation kinetics of real, nonideal mixtures show deviations from this simple rule.^13−15^ For example, the evaporation rate *k*_H_2_O–Ethanol_(*x*_H_2_O_) of the water–ethanol mixture at a water concentration of *x*_H_2_O_ is higher than the rate predicted from the linear combination of the rates of pure water (*k*_H_2_O_) and pure ethanol (*k*_Ethanol_): *k*_H_2_O–Ethanol_(*x*_H_2_O_) > *x*_H_2_O_*k*_H_2_O_ + (1 – *x*_H_2_O_)*k*_Ethanol_, where *x*_H_2_O_ denotes the mole fraction of water. In turn, the water–formic acid mixture shows the opposite trend (Figure 1a, see the Supporting Information for more details). Although these differences in the evaporation rates largely reflect the evaporation energetics (i.e., the respective vapor pressures as shown in Figure 1b),^16^ the impact of the different intermolecular interactions in the bulk liquid on the molecular conformation and ordering at the interface of these binary mixtures—highly relevant to evaporation kinetics—has been poorly understood. Figure 1 illustrates that the nonideal behavior is complex: not only do the mixtures exhibit different deviations from ideality, but there is also a strong asymmetry with respect to the molar composition. For instance, for the water–ethanol mixture, the maximum deviation of the vapor pressure occurs at *x*_H_2_O_ = 0.85, while the excess evaporation rate peaks at lower values of *x*_H_2_O_. The following question presents itself: What is happening at the surface of the liquid mixture?

Here, we explore the molecular conformations of aqueous binary mixtures using surface-specific vibrational sum-frequency generation (SFG) spectroscopy. This technique can uniquely probe molecules within a few surface layers through their vibrational response.^17,18^ In fact, SFG spectroscopy has already been applied to probe the interfacial structure of various binary liquid mixtures, including water–methanol,^19−23^ water–ethanol,^21,24,25^ and water–formic acid^26^ mixtures probing the O–H stretching mode. However, because these organic species commonly contain O–H groups, SFG spectra in the O–H stretching mode region cannot distinguish both molecular species individually. This challenge can be circumvented by probing the water bending mode, which allows disentangling the water contribution from other vibrational modes such as the C–O–H bending mode of the organic species.^27,28^ However, for aqueous alcohol or formic acid mixtures, the water structure at the interface has not been isolated.

By combining SFG spectroscopy with molecular dynamics (MD) simulations, we determine the interfacial structure and correlate the interfacial structure with evaporation thermodynamics and kinetics for binary mixtures of water with methanol/ethanol/formic acid. We found that the variations of the C–H stretching mode of organic species and the free O–H stretching mode of water upon the addition of the organic species do not show a significant difference between the aqueous binary mixtures of methanol, ethanol, and formic acid. In contrast, the H–O–H bending mode of water in these mixtures differs substantially. The spectra and MD simulation results reveal that water’s interfacial alignment becomes randomized upon adding ethanol and methanol to water, while it becomes ordered upon adding formic acid. Our results thus show that the nonideal interaction in bulk is reflected in the structure of water at the interface, relevant to the evaporation rates.

## II Methods

### II.A Sample Preparation

The mixtures were prepared using methanol (HPLC, gradient grade, ≥99.8% (GC), <0.01% water, VWR International), ethanol (Puriss., absolute, ≥99.8% (GC), Sigma-Aldrich), formic acid (Suprapur, 98–100% (ACS), Merck KGaA), D_2_O (99.9 atom % D, Sigma-Aldrich), and Milli-Q water (18.2 MΩ cm). All substances were used without further purification. To avoid the overlap between the C=O stretching mode (∼1720 cm^–1^)^26^ and the H–O–H bending mode (∼1650–1750 cm^–1^),^28^ we measure the H–O–D bending region for the water–formic acid system. We prepared samples using H_2_O:D_2_O mixtures and formic acid. We used 1:1 H_2_O:D_2_O mixtures for samples with formic acid mole fractions of 0, 0.02, 0.08, 0.14, and 0.32. 1:3 H_2_O:D_2_O mixtures and pure D_2_O were used for formic acid mole fractions of 0.53 and 0.66, respectively. The determination of *x*_HOD_ is given in the Supporting Information. The aqueous binary mixtures were contained in a trough with a diameter of 5 cm for the SFG measurements.

### II.B SFG Measurements

The SFG measurements for the free O–H stretching mode and H–O–H/H–O–D bending mode of all of the aqueous binary mixtures as well as the C–H stretching mode of water–methanol mixtures used a femtosecond Ti:sapphire amplified laser system (Coherent Libra, ∼800 nm, ∼50 fs, 1 kHz) with 5 W output power. We used 2 W to pump an optical parametric amplifier (TOPAS, light conversion) with a non-collinear difference frequency generation stage to generate a broadband IR pulse. Another 1 W of the laser output was passed through an etalon to generate a narrowband visible pulse (∼20 cm^–1^). The incident angles of the visible and IR beams were 64 and 40° with respect to the surface normal, respectively. The visible (20 μJ) and IR (1 μJ) pulses were overlapped spatially and temporally at the sample position. Subsequently, the generated SFG signal was dispersed in a spectrometer (Shamrock 303i, Andor Technology) and detected by an EMCCD camera (Newton, Andor Technology).

For the C–H stretching mode of water–ethanol and water–formic acid mixtures, the experiments were carried out with a femtosecond Ti:sapphire amplified laser system (Spitfire Ace, Spectra-Physics, ∼800 nm, ∼40 fs, 1 kHz) with 5 W output power. The visible (13 μJ) and IR (5 μJ) incident angles were 36 and 41°, respectively. The generated SFG pulse was subsequently guided into a spectrograph (Acton SP 300i, Princeton Instruments) and detected with an EMCCD (Newton, Andor Technology).

The C–H stretching mode and the free O–H stretching mode signals were recorded twice for 3 min at the air–aqueous binary mixture interfaces. For water–alcohol mixtures, we measured the H–O–H bending mode signals twice for 10–15 min at the air–solution interface. For water–formic acid mixtures, the H–O–D bending mode was measured in a similar manner to the H–O–H bending mode. To avoid IR absorption of water vapor, we purged N_2_ for 10 min for the measurements of the free O–H stretching mode and the H–O–H/H–O–D bending mode. All spectra were collected in the ssp (denoting s-, s-, and p-polarized SFG, visible, and IR, respectively) polarization combination. The spectra for the air–binary mixture interfaces were normalized to the non-resonant signal taken from *z*-cut quartz after subtracting a background spectrum.

### II.C MD Simulation Protocols

We carried out the MD simulation for the air–aqueous binary mixture interfaces in the slab geometry. We used the GROMACS software for performing the MD simulations.^30^ For the aqueous binary mixture, we used the OPLS-AA force field model^31^ for methanol and ethanol, the modified OPLS-AA model for formic acid, and the TIP4P/2005 model for water.^32^ The simulation cell was 26.6 Å × 26.6 Å × 350 Å with the periodic boundary condition. We obtained more than 100 ns trajectories, from which we computed the orientation of water in aqueous binary mixtures. The details of the simulation can be found in the Supporting Information.

We evaluated the orientations of the water molecules located in the interfacial region. The interfacial region is defined as |*z* – *z*_G_| < *a*, where *z* is the *z*-coordinate of the oxygen atom of a water molecule, *z*_G_ is the position of the Gibbs dividing surface for the mixtures, and *a*^3^ represents the average volume occupied by a molecule in the liquid phase. Here, the *z*-axis forms the surface normal.

## III Results and Discussion

### III.A Variations of the C–H and Free O–H Stretching Modes at the Air–Binary Mixture Interfaces

**Figure 2:**
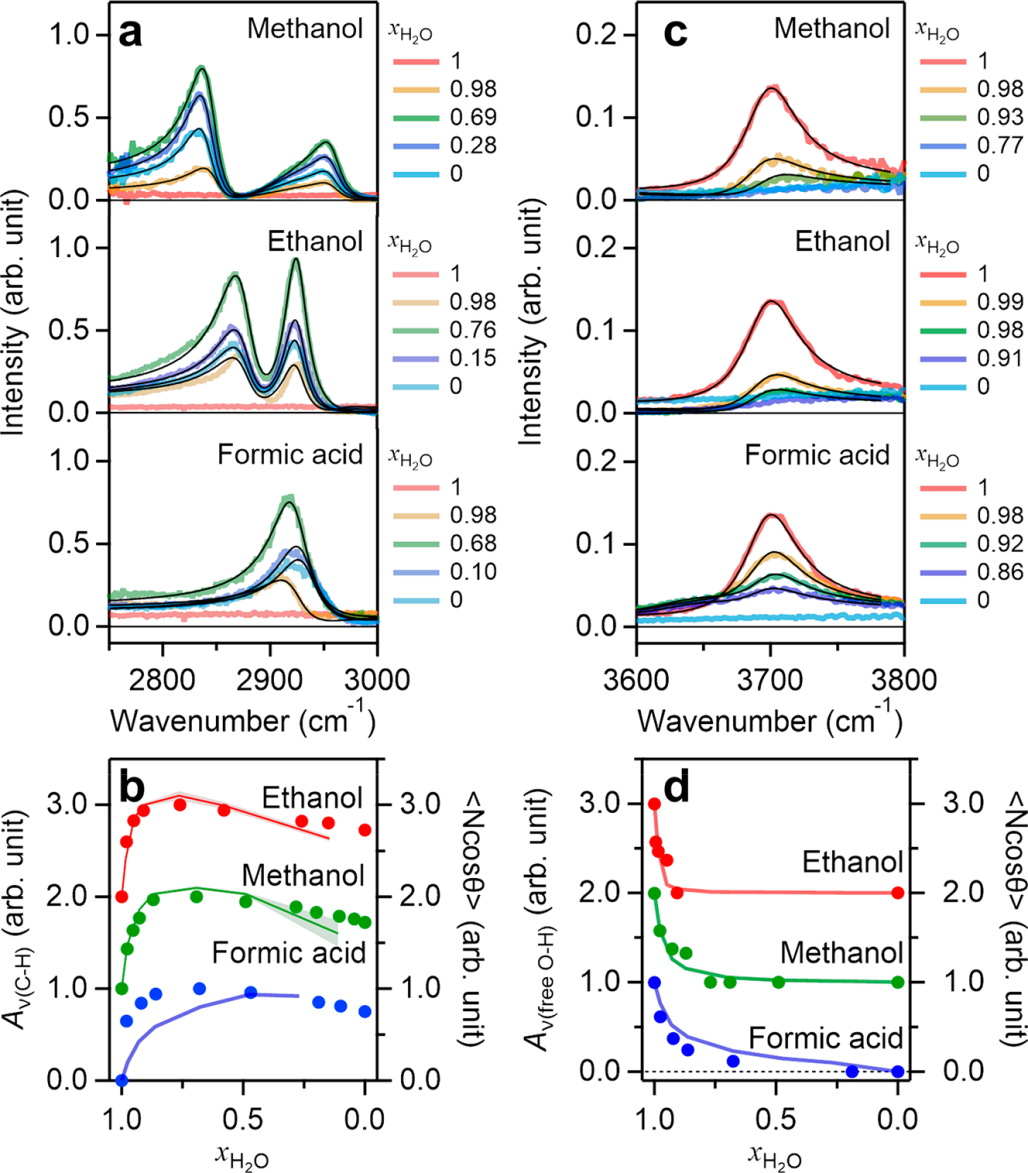
(a) The C–H stretching mode SFG spectra at the air–aqueous binary mixture of methanol, ethanol, and formic acid interfaces. The black lines represent the fits to the SFG spectra. (b) The variations of the amplitudes for the C–H symmetric stretching mode peak of methanol and ethanol CH_3_ group as well as for the C–H stretching mode peak of formic acid together with the simulation data of integrated ⟨*N* cos *θ*⟩ (solid lines). (c) The free O–H stretching mode SFG spectra at the air–aqueous mixture interfaces. The black lines represent the fits to the SFG spectra. (d) The variation of *A*_*ν*(free O–H)_ with composition together with the simulation data of integrated ⟨*N* cos *θ*⟩ (solid lines). Data for different mixtures in parts b and d are normalized to the maximum amplitude and offset by increments of 1 for clarity.

We explore the interfacial organization of the organic components at the air–aqueous binary mixture interfaces by probing the C–H stretching mode ν(C–H) of methanol/ethanol/formic acid with SFG spectroscopy. The measured SFG signal is determined by the average molecular orientation and proportional to the number of interfacial molecules contributing to the signal. Figure 2a displays the ν(C–H) SFG spectra with various mixture concentrations. All of the data except for pure H_2_O show clear C–H stretching mode peaks, reflecting that the hydrophobic groups of the topmost methanol/ethanol/formic acid molecules point out of the liquid phase. The SFG spectra at the air–aqueous binary mixture of the methanol interface show peaks at 2840 and 2960 cm^–1^ and a shoulder at 2930 cm^–1^, which are assigned to the symmetric C–H stretching mode, the antisymmetric C–H stretching mode, and the Fermi resonance of the C–H stretching and C–H bending modes, respectively.^33−35^ Despite earlier assignments to the Fermi resonance,^20^ a recent study provides evidence for the ∼2960 cm^–1^ band to be due to the antisymmetric CH_3_ stretching mode.^33^ For the interface of the water–ethanol mixtures, the 2876 cm^–1^ Fermi resonance and the 2924 cm^–1^ symmetric C–H stretching mode of the CH_3_ group govern the SFG spectra in the C–H range,^36^ while the 2970 cm^–1^ antisymmetric C–H stretching mode and the C–H stretching mode of the CH_2_ groups are weak—in line with a previous study.^20^ At the interface of the aqueous binary mixture of formic acid with air, a single peak is observed at 2930 cm^–1^, which arises from the C–H stretching mode of formic acid.^26^ The frequency shift of the C–H stretching peak with varying water concentration is attributed to cis-/trans-conformations of the formic acid.^37^

To quantify the changes in the spectra, we fit the data with a Lorentzian line shape model, and the obtained fit curves are represented by solid black lines in Figure 2a. We plot the inferred amplitude of the symmetric C–H stretching mode of the CH_3_ group for methanol/ethanol and the C–H stretching mode for formic acid (*A*_*ν*__(C–H)_) in Figure 2b (see the Supporting Information). All mixtures have in common that the C–H stretching mode contribution increases steeply with decreasing *x*_H_2_O_ from 1 to ∼0.7, while it starts to decrease when *x*_H_2_O_ decreases below 0.7. The turnover behavior of *A*_*ν*__(C–H)_ is consistent with previous studies,^19,21,38−40^ indicative of randomization of the C–H groups,^22^ with decreasing water concentration in the binary mixtures.

We then investigated the structure of the topmost O–H groups. We measure the free O–H stretching mode (*v*(free O–H)) SFG features at the air–aqueous binary mixture interfaces, which allows quantifying the number of non-hydrogen-bonded, free O–H groups at the interface. We note that these experiments require purging with N_2_ to exclude water vapor, yet the effect of purging on the C–H stretching mode is negligible (Supporting Information) and our spectra reflect the equilibrium interfacial composition. The measured spectra displayed in Figure 2c show that the free O–H stretching peak is centered at 3700 cm^–1^ ^41^ for all mixtures and vanishes quickly upon the addition of methanol/ethanol/formic acid. To quantify these trends, we determine the amplitudes of the free O–H peak, *A*_*ν*__(free O–H)_ (Figure 2d and the Supporting Information). These amplitudes indicate that the free O–H contribution decreases rapidly with decreasing *x*_H_2_O_ and approaches zero already for *x*_H_2_O_ = 0.85. Also, this observation is consistent with previous studies.^26,42^

The variations of the C–H and free O–H stretching modes are consistent with MD simulation data. From the MD result, we calculated the angle *θ* of the transition dipole moment orientation of symmetric C–H stretching modes of methanol and ethanol as well as the C–H stretching mode of formic acid with respect to the surface normal. The MD simulations also provide access to the number of (symmetric) C–H or O–H stretching chromophores *N*. The SFG spectral amplitude is approximately proportional to ⟨*N* cos *θ*⟩, allowing us to compare the simulation data with the experimental data. Parts b and d of Figure 2 show ⟨*N* cos *θ*⟩ for, respectively, the symmetric C–H stretching modes and the free O–H chromophores. The good agreement between the simulation and experiment in Figure 2b and d supports the notion that the turnover behavior of the variations of the C–H and steep decrease of free O–H stretching contributions arise from the interfacial structure change of the binary mixtures upon the addition of methanol/ethanol/formic acid.^22^

The decrease in the free O–H stretching mode contribution together with the increase in the C–H stretching mode contribution with decreasing *x*_H_2_O_ suggests that the addition of the organic solutes results in hydrogen-bond formation of the organic solute to water’s free O–H groups already at very low solute concentrations in the aqueous binary mixtures. Remarkably, the variations of the two molecular groups sticking out of the interfaces, the C–H groups and the free O–H group, behave very similarly for all three aqueous binary mixtures, irrespective of their very different excess mixing properties and the different excess evaporation rates for water–ethanol and water–methanol mixtures compared to water–formic acid mixtures. As such, based on these observations, the interfacial structures as determined from the groups pointing toward air appear similar for all three mixtures. However, the question of whether the different mixing behavior is reflected in the hydrogen-bonded structure of water at the interface remains.

### III.B Orientation of the Interfacial Water Molecules Viewed through the Water Bending Mode

To study interfacial hydrogen-bonded water, one may consider using SFG spectroscopy to probe the hydrogen-bonded O–H stretching mode region. However, as noted in the Introduction, the SFG signal from the O–H stretching modes of hydrogen-bonded water molecules cannot be disentangled from the signal from the O–H groups of methanol/ethanol/formic acid (see the Supporting Information). Moreover, vibrational couplings markedly alter the line shape of the O–H stretching band.^43^ In contrast, the H–O–H bending mode signal arises solely from water and contains no contributions from methanol/ethanol/formic acid. What is more, the line shape of the water bending mode is rather insensitive to intermolecular coupling,^27,44^ making it ideal for probing interfacial water.^45^

**Figure 3:**
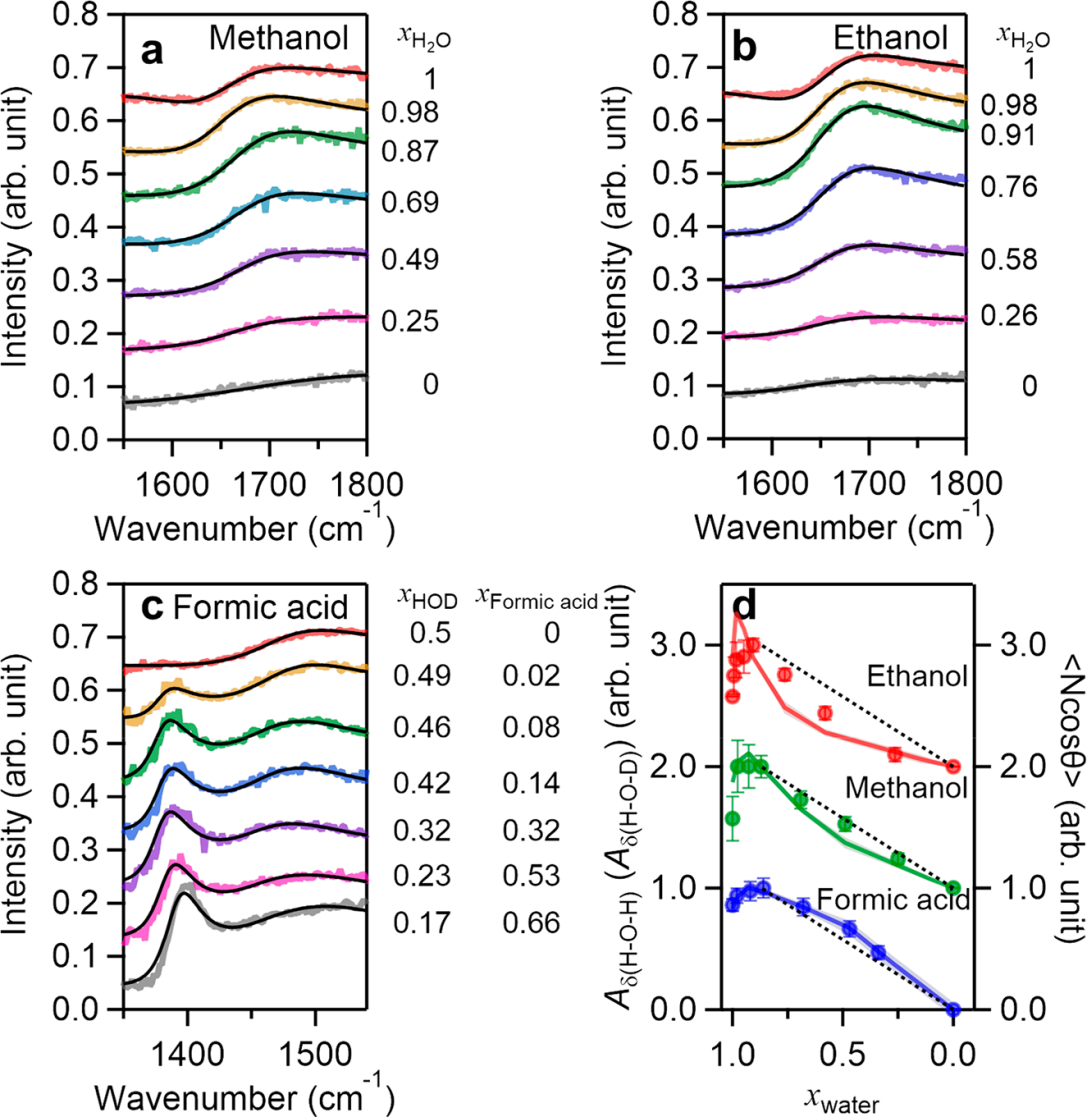
(a–c) SFG spectra at the air–aqueous binary mixture of (a) methanol, (b) ethanol, and (c) formic acid interfaces. Spectra are offset by 0.1 for clarity. The black lines represent the fits to the SFG spectra. (d) *A*_δ(H–O–H)_ for the water–methanol and water–ethanol mixtures together with *A*_δ(H–O–D)_ for the water–formic acid mixture. *A*_δ(H–O–H)_ and *A*_δ(H–O–D)_ were normalized to the values at *x*_H_2_O_ of ∼0.85, where the free O–H stretching mode contributions (*A*_ν(free O–H_)) become vanishingly small. For mixtures with formic acid, *x*_water_ corresponds to the fraction of all water species (*x*_H_2_O_ + *x*_HOD_ + *x*_D_2_O_). The error bars are obtained from the fits. The solid lines indicate the simulation data of integrated ⟨*N* cos θ⟩; the dashed lines connect the extrema of the measurements at *x*_H_2_O_ = 0 and 0.85. Data for different mixtures are offset by increments of 1 for clarity.

In Figure 3a and b, we show the measured H–O–H bending mode (δ(H–O–H)) spectra of H_2_O–methanol and H_2_O–ethanol mixtures, respectively. The spectrum for the air–neat water interface (*x*_H_2_O_ = 1) shows a dip at ∼1612 cm^–1^ and a broad bending mode band at 1650–1750 cm^–1^.^28,46−48^ These spectral features are assigned, respectively, to up-oriented water molecules donating one hydrogen bond (the other O–H is free) and to down-oriented water molecules donating two hydrogen bonds (termed bend 1 and bend 2 in the Supporting Information). For mixtures with formic acid, we use the H–O–D bending mode of H_2_O/D_2_O mixtures rather than the H–O–H bending mode because the C=O stretching mode (∼1720 cm^–1^)^26^ partly overlaps and interferes with the H–O–H bending mode (∼1660 cm^–1^) (see the Supporting Information). Figure 3c displays the H–O–D bending mode (δ(H–O–D)) spectra of the isotopically diluted water–formic acid mixture. The H–O–D bending mode frequency is red-shifted by ∼200 cm^–1^ compared to the H–O–H bending mode frequency.^27^ When formic acid is added to water, the C–H bending mode (∼1350–1400 cm^–1^)^49−52^ and the C–O–H bending mode (∼1490 cm^–1^)^53,54^ appear as features in this frequency region together with the H–O–D bending mode contribution (∼1460 cm^–1^).

We extracted the composition-dependent amplitudes by fitting a Lorentzian model (see the Supporting Information) using two bands to the bending mode data. These two bands have been attributed to water molecules that form one hydrogen bond and the other O–H group being a free O–H group and to water molecules whose O–H groups are both pointing into the liquid and are both hydrogen-bonded. Figure 3d displays the variation of the sum of these amplitudes for the H–O–H and H–O–D bending mode amplitudes (*A*_δ(H–O–H)_ and *A*_δ(H–O–D)_). As already apparent from the raw data, the water response initially increases with increasing concentration of the organic solute, showing a maximum at *x*_H_2_O_ = 0.85, after which the band decreases in intensity. The initial increase coincides with the disappearance of the free O–H stretching contribution at *x*_water_ between 1.0 and ∼0.85 (Figure 2d). As such, the initial increase can be ascribed to an increase in the number of doubly H-bond donating water molecules at the interface: the water molecules with a free O–H group reorient to have both O–H groups pointing into the bulk liquid.

Unlike the data in Figure 2, which showed similar behavior for the three mixtures, the variation of *A*_δ(H–O–H)_ and *A*_δ(H–O–D)_ with composition differs markedly for *x*_water_ < 0.85 for the three mixtures. To better illustrate these differences, we normalize the bending mode amplitudes in Figure 3d to the maximum amplitude at *x*_water_ = 0.85 at which the free O–H contribution has vanished and the associated rotation of water molecules hardly affects the bending mode amplitudes and connect that to the zero signal at *x*_water_ = 0 (dashed lines in Figure 3d). For the water–methanol and water–ethanol mixtures, *A*_δ(H–O–H)_ is, respectively, moderately and significantly below this line; for the water–formic acid mixture at *x*_water_ < 0.85, the results lie above the line (Figure 3d). As such, our results demonstrate positive (negative) excess spectral amplitude for the water bending vibration in mixtures with formic acid (methanol and ethanol) at *x*_water_ < 0.85. These excess spectral amplitudes of water correlate with the evaporation energetics and kinetics: Mixtures exhibiting a positive excess evaporation rate and vapor pressure exhibit reduced spectral amplitudes.

The negative and positive excess spectral amplitudes for the bending mode are indicative of different degrees of ordering of interfacial water for the different mixtures, with the larger signal for formic acid implying more ordered water. We test this hypothesis by performing MD simulations and calculate the angle *θ* of the H–O–H angular bisector with respect to the surface normal for hydrogen-bonded water molecules at the interface. By calculating the integrated ⟨*N* cos *θ*⟩ along the surface normal, where *N* is the number of water molecules, one can compare the simulation data of ⟨*N* cos *θ*⟩ with the experimental data of *A*_δ(H–O–H)_ or *A*_δ(H–O–D)_. The result is shown as solid lines in Figure 3d, demonstrating good agreement with the experimental data. Clearly, the MD simulations capture the variation of the interfacial water response.

**Figure 4:**
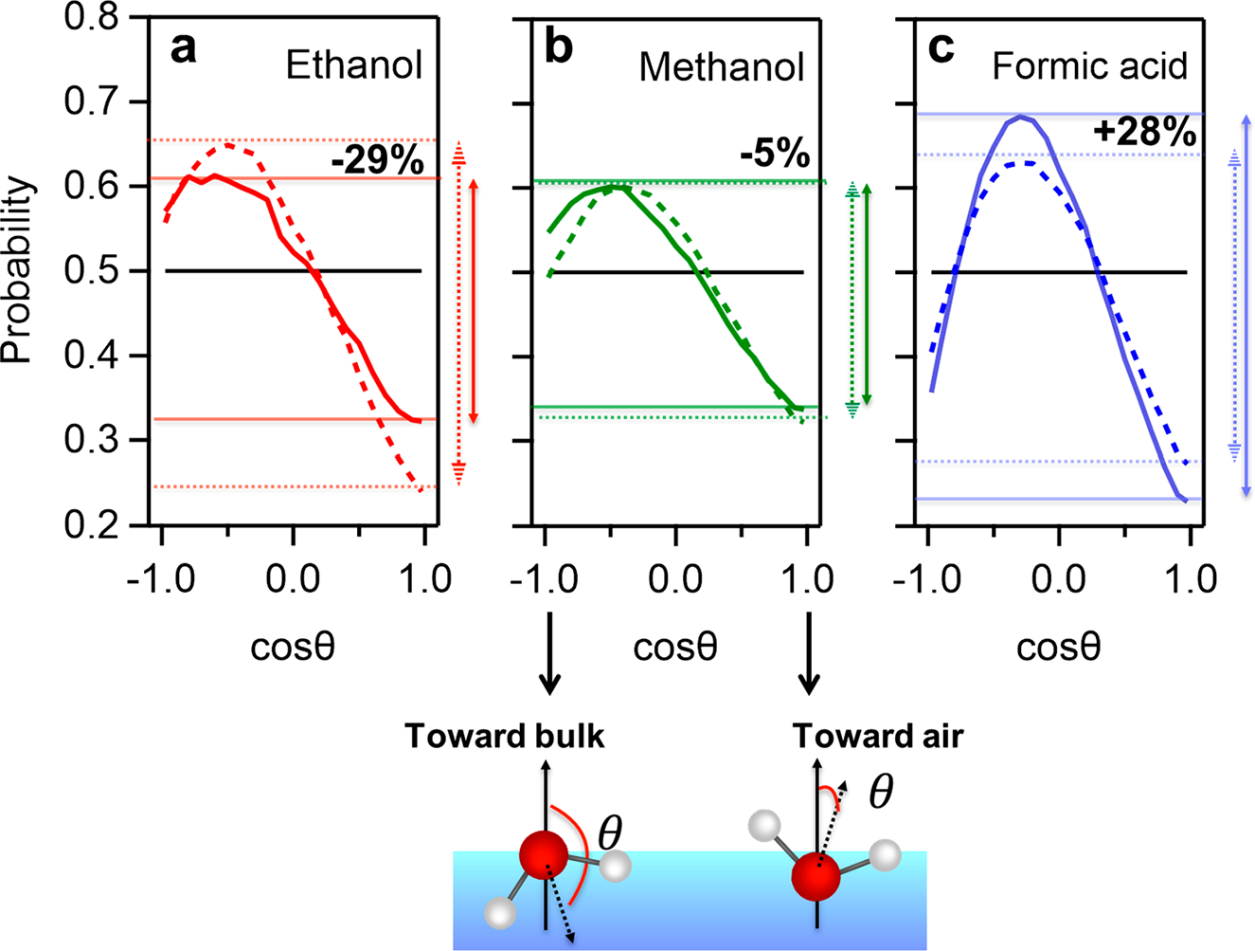
(a–c) The cos *θ* distribution probability of the angular H–O–H bisector of the interfacial water for *x*_H_2_O_ = 0.5 (solid lines) and 0.85 (dotted lines) for the aqueous binary mixtures of (a) ethanol, (b) methanol, and (c) formic acid. The horizontal black lines reflect a totally randomized distribution; the arrows indicate the variations of maximum (Max(*P*_*x*_H2O__)) and minimum (Min(*P*_*x*_H2O__)) ranges of the cos *θ* distribution. The percentages given in the figures indicate the relative change of these ranges, [(Max(*P*_0.5_) – Min(*P*_0.5_)) – (Max(*P*_0.85_) – Min(*P*_0.85_))]/[Max(*P*_0.85_) – Min(*P*_0.85_)].

The reduced (increased) ⟨*N* cos *θ*⟩ values (and spectral amplitudes *A*_δ(H–O–H)_ or *A*_δ(H–O–D)_) may stem from a reduced (increased) interfacial water density (*N*) or reduced (enhanced) water alignment (⟨cos *θ*⟩). Our MD simulation data show that the variations of the spectral amplitudes are caused by reduced (enhanced) water alignment: At *x*_H_2_O_ ∼ 0.85 (dotted line in Figure 4a–c), where rotation due to hydrogen-bond formation of initially free O–H groups is completed (see the discussion above), the widths of the distributions of ethanol and formic acid are similar. However, for ethanol, it is centered at larger angles (⟨cos *θ*⟩ = −0.5), as compared to formic acid (⟨cos *θ*⟩ = −0.3). The orientational distribution in mixtures with methanol is much broader and centered at ⟨cos *θ*⟩ = −0.4. These trends are enhanced upon increasing the solute concentration to *x*_H_2_O_ ∼ 0.5: For the ethanol sample, the cos *θ* distribution of the angular H–O–H bisector direction broadens upon the addition of ethanol, which means that the orientation of water is further randomized. The distributions for methanol are similar to ethanol, yet the variation of the distribution upon increasing methanol content is smaller than that for ethanol. In contrast to the methanol/ethanol samples, the distribution of water orientations in the presence of formic acid becomes more narrow upon increasing the formic acid concentration. As such, the simulation results demonstrate an enhanced alignment of water upon mixing with formic acid, slightly disordered water for methanol, and substantially disordered water for ethanol.

Our results, therefore, show that the different excess mixing properties in the bulk liquids result in markedly different structuring of water for these mixtures at the interface. The deviations from Raoult’s law point to stronger (weaker) solute–water interactions as compared to water–water interactions for formic acid (methanol and ethanol), leading to stronger (weaker) constraints on the orientation of water. Interestingly, the stronger solute–water interactions for formic acid lead to enhanced ordering of interfacial water, and the sample shows reduced evaporation rates. Inversely, when the degree of the surface organization is reduced, i.e., the surface structure becomes more randomized, as is seen upon the addition of ethanol to water, the sample shows an excessive evaporation rate.

## IV Conclusion

We performed surface-specific vibrational SFG spectroscopy and MD simulations for the air–aqueous binary mixture interfaces of ethanol, methanol, and formic acid to explore their surface molecular conformations. Despite the largely differing excess mixing properties of the three studied mixtures, the variation of the outermost arrangement judged by the presence of spectral signatures of free O–H groups and the C–H stretching mode is similar for all three mixtures. On the other hand, we find different ordering of water molecules as determined from water’s bending mode vibrations: Our SFG and MD simulation data show that the difference of excess mixing properties also propagates to the interfacial structure of the one to two topmost layers of the binary mixtures; reduced (enhanced) intermolecular interactions and accelerated (reduced) evaporation rates relative to ideal evaporation are linked with a lesser (greater) surface reorganization of the interfacial water molecules. The bending mode provides us new information about the complex interfacial structure, which paves a path for understanding evaporation and condensation processes, and ultimately perhaps a pathway toward controlling evaporation processes.

## Supporting Information Available

Evaporation measurement details, all SFG spectra and fitting procedures, estimation of HOD mole fraction, and MD simulation details (PDF)
