# Supplementary material for: Interfacial Water Structure of Binary Liquid Mixtures Reflects Nonideal Behavior
Source: J Phys Chem B. 2021 Sep 10;125(37):10639–46. doi: 10.1021/acs.jpcb.1c06001 (PMC8474108; doi:10.1021/acs.jpcb.1c06001)
Supplement: Supplementary file 1 — jp1c06001_si_001.pdf [file jp1c06001_si_001.pdf]

Supporting Information: Interfacial Water Structure of Binary Liquid Mixtures Reflects Non-Ideal Behavior

Xiaoqing Yu,[1,#] *Takakazu Seki,*[1,#] *Chun-Chieh Yu,*[1] *Kai Zhong,*[2] *Shumei Sun,*[3] *Masanari Okuno,*[4] *Ellen H. G. Backus,*[1,5] *Johannes Hunger,*[1] *Mischa Bonn,*[1,*] *and Yuki Nagata*[1,*]

1. Max Planck Institute for Polymer Research, Ackermannweg 10, 55128 Mainz, Germany

2. University of Groningen, Zernike Institute for Advanced Materials, Nijenborgh 4, 9747 AG Groningen, the Netherlands

3. Department of Physics, Applied Optics Beijing Area Major Laboratory, Beijing Normal University, 100875 Beijing, China

4. Department of Basic Science, Graduate School of Arts and Sciences, The University of Tokyo, Komaba, Meguro, 153-8902 Tokyo, Japan

5. Department of Physical Chemistry, University of Vienna, Währinger Strasse 42, 1090 Vienna, Austria

[*]Correspondence: bonn@mpip-mainz.mpg.de, nagata@mpip-mainz.mpg.de

[#]These authors contributed equally

Table of Contents

1. Evaporation Rate Measurement 3

2. Estimation of Vapor Pressure of Formic Acid at 333 K 4

3. Relative Vapor Pressure Calculation 5

4. N_2 Purging Effect on the SFG Measurements 5

5. Fitting Procedure for SFG Spectra of C-H Stretching Mode at the Interfaces of the Air-Aqueous Binary Mixture 6

6. Fitting Procedure for SFG Spectra of Free O-H Stretching Mode at the Interfaces of the Air-Aqueous Binary Mixture 13

7. SFG Spectra of the Bonded O-H Stretching Mode at the Interfaces of the Air-Aqueous Binary Mixture of Methanol 17

8. Fitting Procedure for SFG Spectra of the Water's Bending Mode at the Interfaces of the Air-Aqueous Binary Mixture 17

9. Estimation of Water Mole Fraction in the Water-Formic Acid Mixture 25

10. MD Simulation Details 26

11. Calculation of $\boldsymbol{N\cos\theta}$ for C-H of the Organic Components and Free O-H of Water..28

12. Depth Profiles of Density and Orientation 29

13. Comparison of Different Force Field Models 31

References 33

1. Evaporation Rate Measurement

We prepared various concentrations of the aqueous binary mixtures samples in a trough with a diameter of 2 cm and total volume 3.5 mL, and placed the samples in a N_2-purged chamber. After the relative humidity in the chamber reached below 10%, we evaluated the evaporation rate by recording the surface height of the samples over time with a height displacement sensor (Keyence Corporation, LK-G85), i.e., the slope of the height variation *vs.* time. The temperature in the chamber was kept at 22.4–22.6 °C during the measurement. The measured evaporation rate ($k_{H_2O-org}(x_{H_2O})$) is displayed in Fig. S1. The dotted lines in Fig. S1 represent the evaporation rate ($k_{pred}(x_{H_2O})$) predicted from the evaporation rate of pure water (k_{H_2O}) and pure organic species(k_{org}): $k_{pred}(x_{H_2O}) = x_{H_2O}k_{H_2O} + (1 - x_{H_2O})k_{org}$.

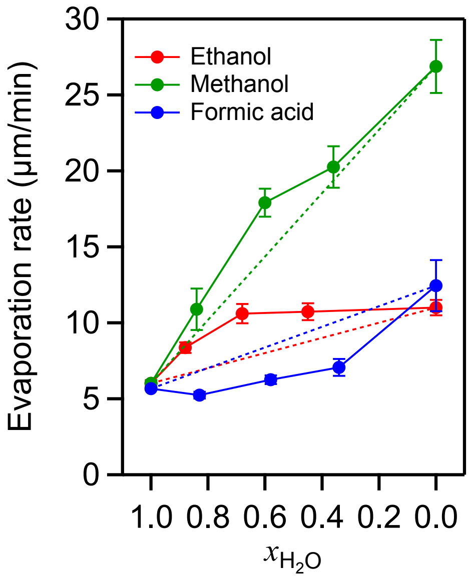

Figure S1. Variations of evaporation rate *vs.* water mole fraction, x_{H_2O}. The error bar indicates the 95% confidence interval of the estimation of the evaporation rate.

2. Estimation of Vapor Pressure of Formic Acid at 333 K

The vapor pressure at 333 K for the water-formic acid mixture was extrapolated from those at 388 K and 398 K in ref 1, using a simple semi-empirical Antoine equation[2,3], $lnp_i = a + b/T$, where p_i represents the vapor pressure corresponding to ith component in the system at the temperature T. Based on the p_i values at 388 K and 398 K, we determined the p_i values at 333 K for the data with various molar fraction of water (x_1) and formic acid (x_2) in the liquid phase. Here, we assume that, upon changing the temperature, the molecular compositions in the gas- and liquid-phases do not vary. These data are displayed in Table S1.

Table S1. The partial vapor pressure of water (p_1) and formic acid (p_2) calculated from Antoine equation and estimated deviations from Raoult's law for p_1.

x_1	p_1 (388 K) (kPa)	p_1 (398 K) (kPa)	a	b	p_1 (333 K) (kPa)	$x_1p_1(x_1=1)$	Deviation (%)
0.00	0.00	0.00	0.00	0.00	0.00	0.00	0.00
0.16	16.95	26.07	19.95	-6643.23	1.00	3.22	-68.91
0.41	50.31	71.79	18.07	-5491.79	4.86	8.11	-40.08
0.53	76.08	107.84	18.22	-5387.11	7.68	10.56	-27.29
0.63	97.43	138.40	18.55	-5419.99	9.70	12.52	-22.52
0.74	124.24	172.98	17.99	-5110.72	14.11	14.75	-4.39
0.85	142.97	198.48	18.02	-5065.41	16.55	16.95	-2.34
0.91	153.17	211.49	17.87	-4982.37	18.37	18.12	1.34
1.00	167.92	232.11	18.01	-4999.37	19.99	19.99	0.00
x_2	p_2 (388 K) (kPa)	p_2 (398 K) (kPa)	a	b	p_2 (333 K) (kPa)	$x_2p_2(x_2=1)$	Deviation (%)
1.00	153.70	200.60	15.63	-4112.47	26.69	26.69	0.00
0.84	121.55	157.23	15.05	-3975.40	22.38	22.39	-0.04
0.59	78.19	104.91	16.06	-4538.55	11.33	15.87	-28.63
0.47	55.02	74.17	15.89	-4610.28	7.73	12.59	-38.60
0.37	38.57	51.60	15.24	-4495.82	5.69	9.98	-42.98
0.26	19.66	27.72	16.65	-5306.22	2.05	6.99	-70.63
0.15	10.73	14.82	15.24	-4993.91	1.28	4.07	-68.51
0.09	6.23	9.11	16.94	-5863.08	0.51	2.49	-79.39
0.00	0.00	0.00	0.00	0.00	0.00	0.00	0.00

3. Relative Vapor Pressure Calculation

Figure S2 shows the total vapor pressure and partial water vapor pressure of aqueous binary mixtures of methanol, ethanol, and formic acid at 333 K. The relative vapor pressure was calculated via, $\Delta p(x_{H_2O}) = p_{H_2O-org}(x_{H_2O})/p_{pred}(x_{H_2O}) - 1$, where $p_{H_2O-org}(x_{H_2O})$ is the real vapor pressure[1,4,5], $p_{pred}(x_{H_2O})$ is the predicted one based on Raoult's law: $p_{pred}(x_{H_2O}) = x_{H_2O}p_{H_2O} + (1 - x_{H_2O})p_{org}$.

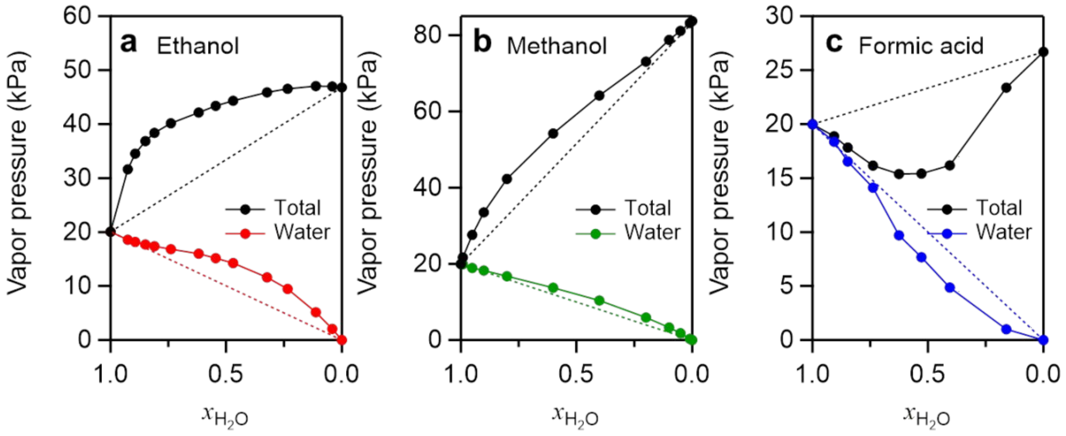

Figure S2. (a-c) Total vapor pressure and partial water vapor pressure of aqueous binary mixtures of (a) ethanol, (b) methanol, and (c) formic acid at 333 K. The dotted lines represent the vapor pressure following Raoult's law. The data for (a) and (b) were obtained from refs 4 and 5, respectively. The data for (c) were extrapolated in the previous section[1] using the Antoine equation.

4. N_2 Purging Effect on the SFG Measurements

It was argued that the evaporation with and without carrier gas might affect the evaporation process by affecting the diffusion of the evaporated molecules[6,7]. As such, purging with N_2 as required for the SFG experiments at the free-OH stretching frequency, the interfacial composition may not be in equilibrium. To explore the effect of N_2 purging on the SFG spectra, we compared the C-H stretching mode signal with and without N_2 purging. Fig. S3

shows that the signals of both 2% methanol and pure methanol samples do not change by using N_2 purging. Therefore, the SFG spectra when purging can be approximated to represent the equilibrium conditions and it is reasonable to compare equilibrium vapor pressures to our SFG results.

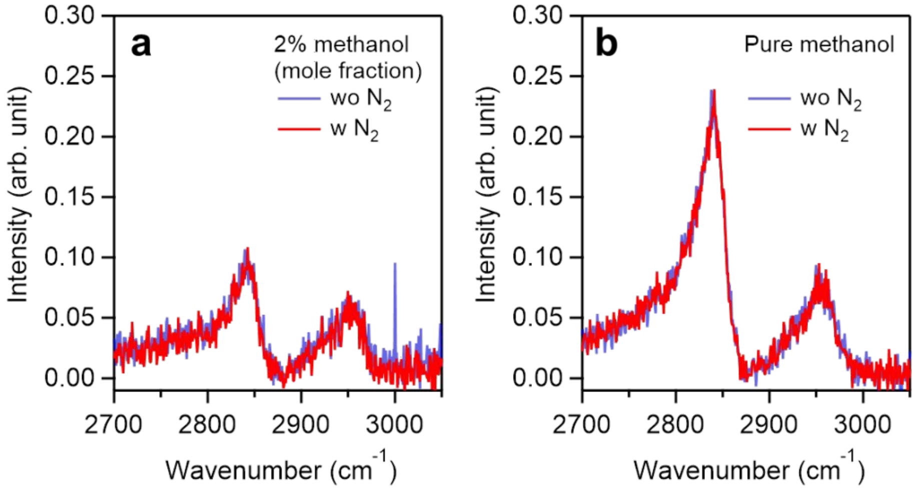

Figure S3. SFG spectra of the C-H stretching mode at the interface of the air-aqueous binary mixture of 2% (a) and pure methanol (b) with and without purging with N_2 gas.

5. Fitting Procedure for SFG Spectra of C-H Stretching Mode at the Interfaces of the Air-Aqueous Binary Mixture

Figure S4 shows the measured SFG spectra at the interface of the air-aqueous binary mixture of methanol. We extracted the amplitude of the symmetric C-H stretching mode of CH_3 group by fitting the SFG spectra of the water-methanol mixtures. The SFG signal ($\chi_{\mathrm{eff}}^{(2)}(\omega)$) consists of the second-order susceptibilities;

$$\left|\chi_{\mathrm{eff}}^{(2)}(\omega)\right|^2 = \left|\chi_{\mathrm{NR}}^{(2)} + \chi_{\mathrm{ss}}^{(2)}(\omega) + \chi_{\mathrm{Fermi}}^{(2)}(\omega) + \chi_{\mathrm{as}}^{(2)}(\omega)\right|^2 \tag{S1}$$

where $\chi^{(2)}_{\mathrm{NR}}$ is non-resonant contribution, $\chi^{(2)}_{\mathrm{ss}}$ and $\chi^{(2)}_{\mathrm{as}}$ are the symmetric and antisymmetric C-H stretching modes of the CH_3 groups[8,9], respectively, and $\chi^{(2)}_{\mathrm{Fermi}}$ represents the Fermi resonance between the symmetric stretching mode and the overtone of the H-C-H bending mode (~1455 cm^{-1}).[8,10] We assumed that the second-order susceptibilities have a Lorentzian lineshape;

$$\chi^{(2)}_{\mathrm{X}}(\omega) = \frac{A_{\mathrm{X}}}{\omega - \omega_{\mathrm{X}} + i\Gamma_{\mathrm{X}}} \tag{S2}$$

where X denotes a vibrational mode, A_{X} and ω_{X} indicate the amplitude and the characteristic frequency of the vibrational mode X, respectively, and $2\Gamma_{\mathrm{X}}$ is the full width at half maximum (FWHM). The fits are shown in Fig. S4, and the obtained fitting parameters are summarized in Table S2.

The ~2950 cm^{-1} peak has often been assigned to a Fermi resonance (Refs. 11–13). This assignment has been done based on polarization-dependent SFG intensities (Ref. 11), assuming a delta function for the orientational distribution function for the CH_3 vibrational chromophores. However, previous MD simulations have shown that the orientational distribution markedly differs from a delta function. Rather, the orientational distribution function decays exponentially.[14] The different orientational distributions also result in different ssp and ppp intensities, as we have shown in our previous work.[15] Based on these considerations, the peak assignment given in Ref. 11 should be reconsidered. Our previous post vibrational self-consistent field calculation for methanol beyond the classical/harmonic vibration approach (Refs. 8 and 9), which can accurately predict Fermi resonances, indicates that the 2950 cm^{-1} contribution can be assigned to the antisymmetric stretching mode of the

CH_3 group. As such, we assign the 2950 cm^{-1} peak to the antisymmetric stretching mode in the present study.

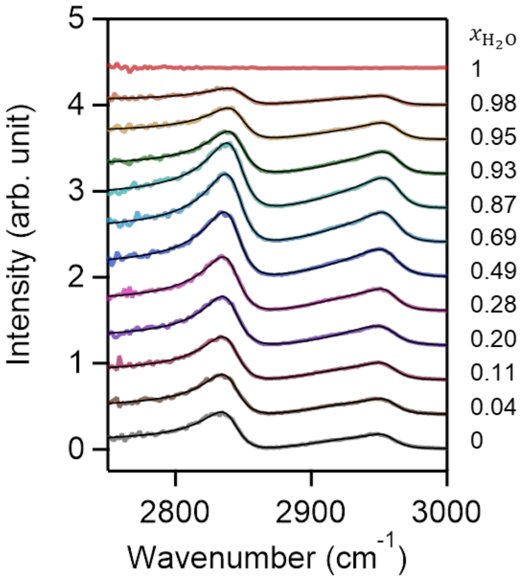

Figure S4. SFG spectra of the C-H stretching mode at the interface of the air-aqueous binary mixture of methanol. The fits with the Lorentzian model are shown as black solid lines. These data are offset by 0.4 for clarity.

Table S2. The fitting parameters for the C-H stretching mode at the interface of the air-aqueous binary mixture of methanol with various water mole fractions, x_{H_2O}.

x_{H_2O}	0.98	0.95	0.93	0.87	0.69	0.49	0.28	0.20	0.11	0.04	0.00
A_{NR}	-0.06	-0.07	-0.07	-0.09	-0.10	-0.09	-0.08	-0.07	-0.08	-0.07	-0.08
Φ_{NR}(rad)	-0.80	-0.80	-0.80	-0.80	-0.80	-0.80	-0.80	-0.80	-0.80	-0.80	-0.80
A_{ss}	1.76	2.60	3.16	3.98	4.10	4.02	3.66	3.54	3.23	3.12	2.96
ω_{ss}(cm^{-1})	2847	2846	2845	2844	2844	2842	2842	2841	2841	2841	2841
Γ_{ss}(cm^{-1})	16	16	16	16	16	16	16	16	16	16	16
A_{Fermi}	7.08	8.87	8.79	10.38	9.85	8.84	7.98	6.90	7.26	6.75	6.97
ω_{Fermi}(cm^{-1})	2941	2942	2940	2939	2935	2931	2929	2925	2928	2927	2929
Γ_{Fermi}(cm^{-1})	36	36	36	36	36	36	36	36	36	36	36
A_{as}	-3.13	-4.05	-3.84	-4.52	-4.19	-3.62	-3.16	-2.69	-2.81	-2.57	-2.69
ω_{as}(cm^{-1})	2957	2959	2961	2960	2959	2958	2958	2957	2957	2957	2956
Γ_{as}(cm^{-1})	15	15	15	15	15	15	15	15	15	15	15

Figure S5 shows the measured SFG data at the interface of the air-aqueous binary mixture of ethanol. Similarly, the SFG spectra of the water-ethanol mixture samples were fitted via;

$$\left|\chi_{\mathrm{eff}}^{(2)}(\omega)\right|^2 = \left|\chi_{\mathrm{NR}}^{(2)} + \chi_{\mathrm{Fermi}}^{(2)}(\omega) + \chi_{\mathrm{ss}}^{(2)}(\omega) + \chi_{\mathrm{as}}^{(2)}(\omega)\right|^2 \tag{S3}$$

by assuming the Lorentzian lineshape expressed by Eq. (S2), where $\chi_{\mathrm{Fermi}}^{(2)}$ represents the Fermi resonance located at around 2876 cm^{-1}, $\chi_{\mathrm{ss}}^{(2)}$ is the symmetric C-H stretching mode of CH_3 groups at around 2925 cm^{-1} [16], and $\chi_{\mathrm{as}}^{(2)}(\omega)$ is the antisymmetric C-H stretching mode at 2970 cm^{-1}.[11,16] The fits are shown in Fig. S5, and the obtained fitting parameters are summarized in Table S3.

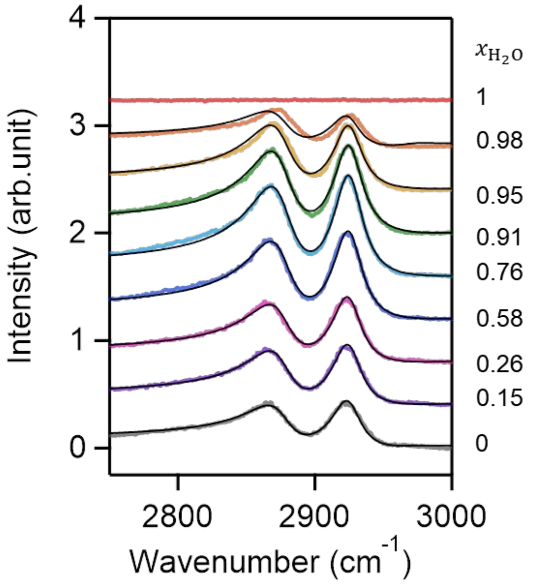

Figure S5. SFG spectra of the C-H stretching mode at the interface of the air-aqueous binary mixture of ethanol. The fits with the Lorentzian model are shown as black solid lines. These data are offset by 0.4 for clarity.

Table S3. The fitting parameters for the C-H stretching mode at the interface of the air-aqueous binary mixture of ethanol with various water mole fractions, x_{H_2O}.

x_{H_2O}	0.98	0.95	0.91	0.76	0.58	0.26	0.15	0.00
A_{NR}	-0.22	-0.22	-0.22	-0.22	-0.22	-0.22	-0.22	-0.22
Φ_{NR}(rad)	-0.36	-0.36	-0.36	-0.36	-0.36	-0.36	-0.36	-0.36
A_{Fermi}	6.13	9.20	10.40	10.83	9.85	8.04	7.81	6.63
ω_{Fermi}(cm^{-1})	2876	2876	2876	2876	2876	2876	2876	2876
Γ_{Fermi}(cm^{-1})	19	19	19	19	19	19	19	19
A_{ss}	6.29	8.68	9.86	10.49	9.87	8.64	8.43	7.62
ω_{ss}(cm^{-1})	2925	2925	2925	2925	2925	2925	2925	2925
Γ_{ss}(cm^{-1})	13	13	13	13	13	13	13	13
A_{as}	-1.88	-1.55	-0.51	0.00	0.00	-0.35	-1.00	-1.66
ω_{as}(cm^{-1})	2970	2970	2970	2970	2970	2970	2970	2970
Γ_{as}(cm^{-1})	16	16	16	16	16	16	16	16

Figure S6 shows the measured SFG data at the interface of the air-aqueous binary mixture of formic acid. The spectra of the water-formic acid mixtures were fitted via;

$$\left|\chi^{(2)}_{\mathrm{eff}}(\omega)\right|^2 = \left|\chi^{(2)}_{\mathrm{NR}} + \chi^{(2)}_{\mathrm{v(C-H)}}(\omega)\right|^2 \tag{S4}$$

by assuming the Lorentzian lineshape expressed by Eq. (S2), where $\chi^{(2)}_{\mathrm{v(C-H)}}$ is the C-H stretching mode peaked at ~2930 cm^{-1}.[17] The fits are shown in Fig. S6. The obtained fitting parameters are summarized in Table S4.

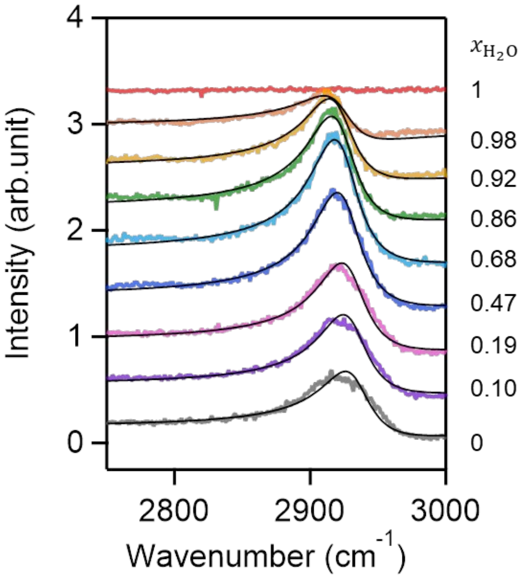

Figure S6. SFG spectra of the C-H stretching mode at the interface of the air-aqueous binary mixture of formic acid. The fits with the Lorentzian model are shown as black solid lines. These data are offset by 0.4 for clarity.

Table S4. The fitting parameters for the C-H stretching mode at the interface of the air-aqueous binary mixture of formic acid with various water mole fractions, $x_{\mathrm{H_2O}}$.

$x_{\mathrm{H_2O}}$	**0.98**	**0.92**	**0.86**	**0.68**	**0.47**	**0.19**	**0.10**	**0.00**
A_{NR}	-0.18	-0.19	-0.19	-0.19	-0.18	-0.17	-0.16	-0.16
Φ_{NR}**(rad)**	-1.08	-1.08	-1.08	-1.08	-1.08	-1.08	-1.08	-1.08
$A_{\upsilon(\mathrm{C\text{-}H})}$	8.91	11.62	12.99	13.80	13.23	11.75	11.18	10.38
$\omega_{\upsilon(\mathrm{C\text{-}H})}$**(cm^{-1})**	2923	2921	2922	2924	2925	2929	2930	2933
$\Gamma_{\upsilon(\mathrm{C\text{-}H})}$**(cm^{-1})**	21	21	21	21	21	21	21	21

6. Fitting Procedure for SFG Spectra of Free O-H Stretching Mode at the Interfaces of the Air-Aqueous Binary Mixture

Figures S7, S8, and S9 show the measured SFG spectra at the interfaces of the air-aqueous binary mixture of methanol, ethanol, and formic acid, respectively. The spectra of the air-aqueous binary mixtures in free O-H region were fitted via;

$$\left|\chi^{(2)}_{\mathrm{eff}}(\omega)\right|^2 = \left|\chi^{(2)}_{\mathrm{NR}} + \chi^{(2)}_{\mathrm{shoulder}}(\omega) + \chi^{(2)}_{\mathrm{v(free\ O-H)}}(\omega)\right|^2$$

(S5)

by assuming that $\chi^{(2)}_{\mathrm{shoulder}}(\omega)$ and $\chi^{(2)}_{\mathrm{v(free\ O-H)}}(\omega)$ have a Lorentzian lineshape (S2). The shoulder peak of water is used for the water-formic acid mixture, while it was not used for water-methanol and water-ethanol mixtures. The fit lines are also plotted in Figs. S7, S8, and S9, and the fitting parameters are summarized in Tables S5, S6, and S7 accordingly.

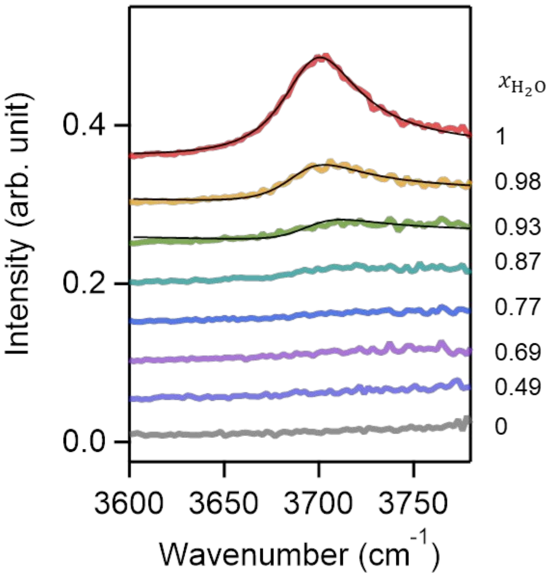

Figure S7. SFG spectra of the free O-H stretching mode at the interface of the air-aqueous binary mixture of methanol. The fits with Lorentzian model are shown as black solid lines. These data are offset by 0.05 for clarity.

Table S5. The spectral fitting parameters for the free O-H stretching mode contribution at the interface of the air-aqueous binary mixture of methanol with various water mole fractions, x_{H_2O}.

x_{H_2O}	**1.00**	**0.98**	**0.93**
A_{NR}	-0.13	-0.12	-0.12
Φ_{NR}**(rad)**	-0.92	-0.23	-0.10
$A_{\upsilon(free\ O\text{-}H)}$	-6.23	-3.61	-2.33
$\omega_{\upsilon(free\ O\text{-}H)}$**(cm^{-1})**	3696	3692	3697
$\Gamma_{\upsilon(free\ O\text{-}H)}$**(cm^{-1})**	25	25	25

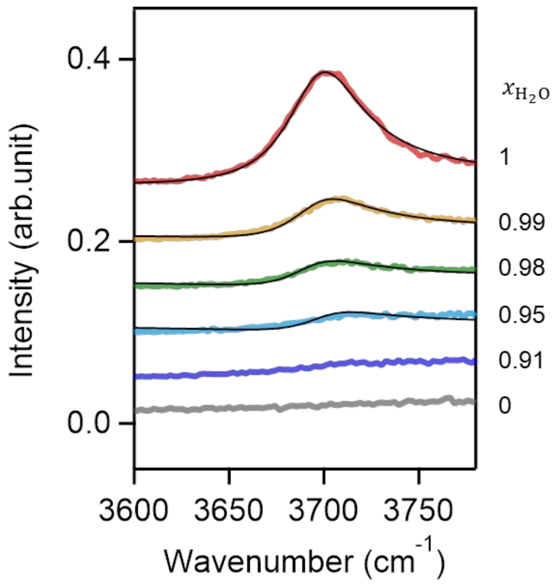

Figure S8. SFG spectra of the free O-H stretching mode at the interface of the air-aqueous binary mixture of ethanol. The fits with Lorentzian model are shown as black solid lines. These data are offset by 0.05 for clarity.

Table S6. The spectral fitting parameters for the free O-H stretching mode at the interface of the air-aqueous binary mixture of ethanol with various water mole fractions, x_{H_2O}.

x_{H_2O}	1.00	0.99	0.98	0.95
A_{NR}	-0.13	-0.10	-0.09	-0.09
Φ_{NR}(rad)	-0.92	-0.32	0.01	0.01
$A_{\upsilon(free\ O\text{-}H)}$	-6.23	-3.56	-2.91	-2.29
$\omega_{\upsilon(free\ O\text{-}H)}(cm^{-1})$	3696	3695	3692	3699
$\Gamma_{\upsilon(free\ O\text{-}H)}(cm^{-1})$	25	25	25	25

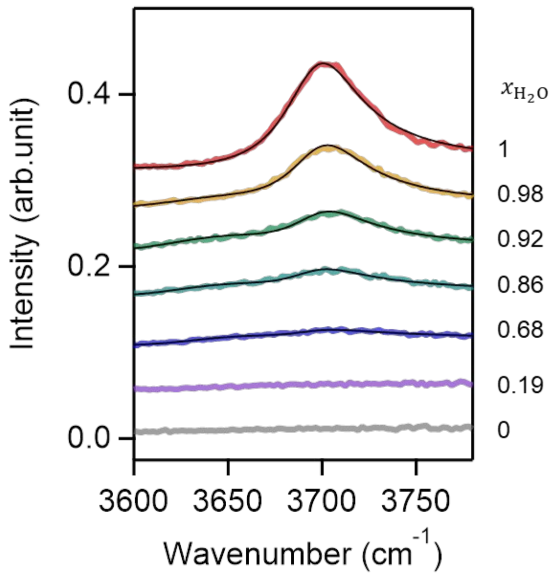

Figure S9. SFG spectra of the free O-H stretching mode at the interface of the air-aqueous binary mixture of formic acid. The fits with Lorentzian model are shown as black solid lines. These data are offset by 0.05 for clarity.

Table S7. The spectral fitting parameters for the free O-H stretching mode at the interface of the air-aqueous binary mixture of formic acid with various water mole fractions, x_{H_2O}.

x_{H_2O}	**1.00**	**0.98**	**0.92**	**0.86**	**0.68**
A_{NR}	-0.13	-0.13	-0.13	-0.13	-0.11
Φ_{NR}**(rad)**	-0.92	-0.92	-0.80	-0.64	-0.14
$A_{\upsilon(\text{free O-H})}$	-6.23	-3.84	-2.31	-1.52	-0.73
$\omega_{\upsilon(\text{free O-H})}$**(cm^{-1})**	3696	3698	3698	3695	3693
$\Gamma_{\upsilon(\text{free O-H})}$**(cm^{-1})**	25	25	25	25	25
A_{shoulder}	0.00	-2.33	-3.35	-2.85	-2.71
ω_{shoulder}**(cm^{-1})**	3640	3640	3640	3640	3640
Γ_{shoulder}**(cm^{-1})**	50	50	50	50	50

7. SFG Spectra of the Bonded O-H Stretching Mode at the Interfaces of the Air-Aqueous Binary Mixtures of Methanol

Figure S10 shows the measured SFG spectra at the interfaces of the air-aqueous binary mixtures with methanol, which display a broad band ranging from 3000 to 3500 cm^{-1}. Difficulties in interpreting the bonded O-H stretching mode spectra arise from the coupling of the O-H stretching band to the bending mode overtone[18,19] and intra- and intermolecular coupling of the stretching chromophores[20]. Therefore, a detailed interpretation of the bonded O-H stretching mode spectra is challenging.

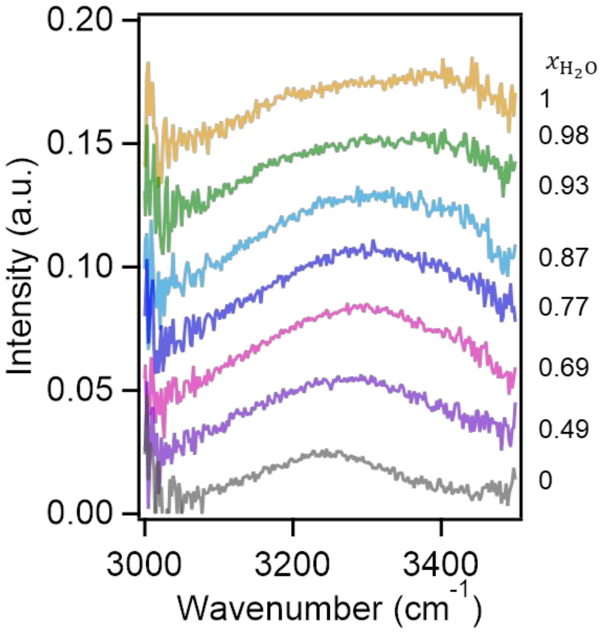

Figure S10. SFG spectra of the O-H stretching mode at the interface of the air-aqueous binary mixture of methanol.

8. Fitting Procedure for SFG Spectra of the Water's Bending Mode at the Interfaces of the Air-Aqueous Binary Mixture

Figure S11 shows the measured SFG data at the interfaces of the air-aqueous binary mixture of methanol and ethanol. We performed the spectral fitting via;

$$\left|\chi_{\mathrm{eff}}^{(2)}(\omega)\right|^2 = \left|\chi_{\mathrm{NR}}^{(2)} + \chi_{\mathrm{bend1}}^{(2)}(\omega) + \chi_{\mathrm{bend2}}^{(2)}(\omega) + \chi_{\mathrm{MeOH/EtOH}}^{(2)}(\omega)\right|^2 \tag{S6}$$

We used two bending modes ("bend1" and "bend2"), which are ascribed to up-oriented water molecules donating one hydrogen bond (the other O-H is the free, non-hydrogen-bonded OH group) and to down-oriented water molecules donating two hydrogen bonds, respectively.[21–24] Based on Ref. [25], we set $\Gamma_{\mathrm{bend1}} = 65\ \mathrm{cm}^{-1}$, $\omega_{\mathrm{bend1}} = 1612\ \mathrm{cm}^{-1}$. We furthermore set $A_{\mathrm{bend1}} \propto A_{\nu(\mathrm{free\ O-H})}$ and assume that $A_{\mathrm{MeOH/EtOH}}$ is proportional to the methanol/ethanol concentration. The phase of the non-resonant contribution (ϕ_{NR}) is independent of their composition. The contribution of δ(H-O-H) in the main text indicates the sum of the A_{bend1} and A_{bend2}.

The obtained fitting parameters of the SFG spectra for methanol and ethanol samples are summarized in Tables S8 and S9.

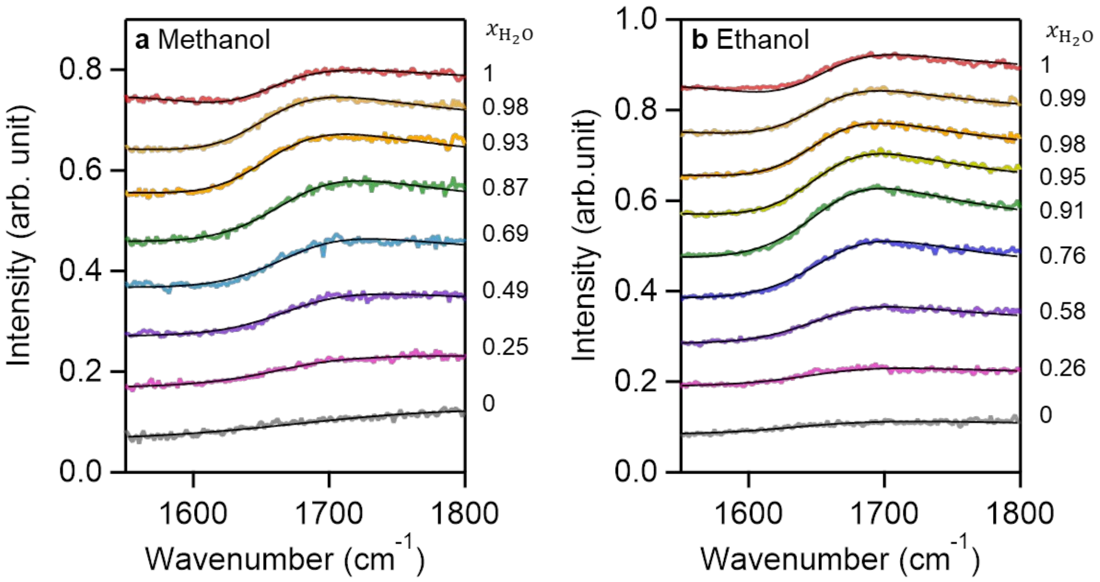

Figure S11. (a-b) SFG spectra in the H-O-H bending frequency region at the interfaces of the air-aqueous binary mixture of methanol and ethanol. The fits with the Lorentzian model are shown as black solid lines. These data are offset by 0.1 for clarity.

Table S8. The spectral fitting parameters for the H-O-H bending mode contribution at the interface of the air-aqueous binary mixture of methanol with various water mole fractions, x_{H_2O}.

x_{H_2O}	**1.00**	**0.98**	**0.93**	**0.87**	**0.69**	**0.49**	**0.25**	**0.00**
A_{NR}	-0.25	-0.27	-0.30	-0.31	-0.32	-0.32	-0.30	-0.29
Φ_{NR}(rad)	-0.12	-0.12	-0.12	-0.12	-0.12	-0.12	-0.12	-0.12
A_{MeOH}	—	-0.20	-1.38	-2.57	-6.13	-10.08	-14.82	-19.77
ω_{MeOH}(cm^{-1})	—	1698	1698	1698	1698	1698	1698	1698
Γ_{MeOH}(cm^{-1})	—	205	205	205	205	205	205	205
A_{bend2}	-11.92	-15.27	-14.28	-12.48	-9.07	-6.58	-3.03	—
ω_{bend2}(cm^{-1})	1659	1657	1660	1673	1674	1676	1670	—
Γ_{bend2}(cm^{-1})	73	73	73	73	73	73	73	—
A_{bend1}	4.77	2.76	1.78	0.00	0.00	0.00	0.00	—
ω_{bend1}(cm^{-1})	1612	1612	1612	1612	1612	1612	1612	—
Γ_{bend1}(cm^{-1})	65	65	65	65	65	65	65	—

Table S9. The spectral fitting parameters for the H-O-H bending mode contribution at the interface of the air-aqueous binary mixture of ethanol with various water mole fractions, x_{H_2O}.

x_{H_2O}	**1.00**	**0.99**	**0.98**	**0.95**	**0.91**	**0.76**	**0.58**	**0.26**	**0.00**
A_{NR}	-0.27	-0.28	-0.29	-0.33	-0.34	-0.35	-0.33	-0.32	-0.30
Φ_{NR}(rad)	-0.22	-0.22	-0.22	-0.22	-0.22	-0.22	-0.22	-0.22	-0.22
A_{EtOH}	—	-0.08	-0.16	-0.40	-0.71	-1.90	-3.33	-5.87	-7.93
ω_{EtOH}(cm^{-1})	—	1637	1637	1637	1637	1637	1637	1637	1637
Γ_{EtOH}(cm^{-1})	—	144	144	144	144	144	144	144	144
A_{bend2}	-11.92	-12.09	-13.23	-13.27	-12.82	-10.06	-6.18	-1.68	—
ω_{bend2}(cm^{-1})	1659	1653	1655	1656	1658	1660	1657	1657	—
Γ_{bend2}(cm^{-1})	65	65	65	65	65	65	65	65	—
A_{bend1}	4.77	2.72	2.23	1.75	0.00	0.00	0.00	0.00	—
ω_{bend1}(cm^{-1})	1612	1612	1612	1612	1612	1612	1612	1612	—
Γ_{bend1}(cm^{-1})	65	65	65	65	65	65	65	65	—

For the aqueous-binary mixture of formic acid, we found that the C=O stretching mode (~1720 cm^{-1}) of formic acid overwhelms the H-O-H bending mode contributions spectrally (Figure S12). To avoid this complication, we measured the H-O-D bending mode with isotopically diluted water-formic acid mixtures. Figure S13 displays the measured

spectra in this frequency region, one can see the C-H bending (~1380 cm^{-1}) and the C-O-H bending mode (~1490 cm^{-1}) features of formic acid.[26,27] Although the band assignment at ~1490 cm^{-1} is controversial, it was reported that the formic acid dimmer in gas phase and crystal forms show C-O-H in-plane bending mode at 1450 and 1560 cm^{-1} respectively.[26] We didn't observe this band for low formic acid concentration, while it's easy to form trans-conformation structure or even dimmer at high concentration. We therefore chose to assign it to the C-O-H bending mode. These modes may slightly change in water-formic acid mixture. Therefore, to determine the modes' parameters further precisely for isotopically diluted water-formic acid system, we performed a global fit for the spectra of isotopically diluted water-formic acid and H_2O-formic acid simultaneously.

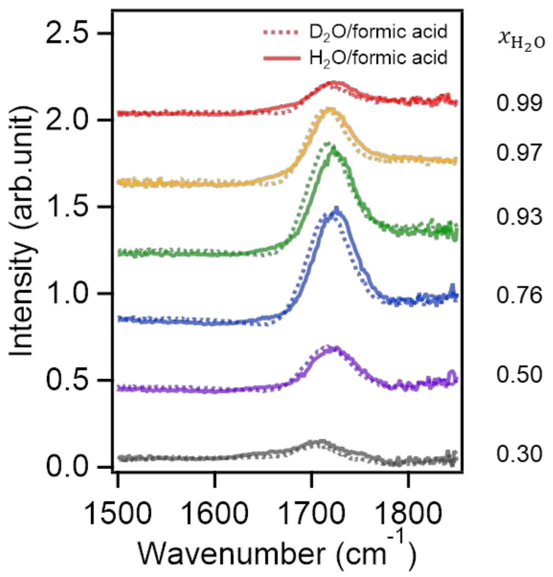

Figure S12. SFG spectra in the H-O-H bending frequency region at the interface of the air-aqueous binary mixture of formic acid. These data are offset by 0.4 for clarity.

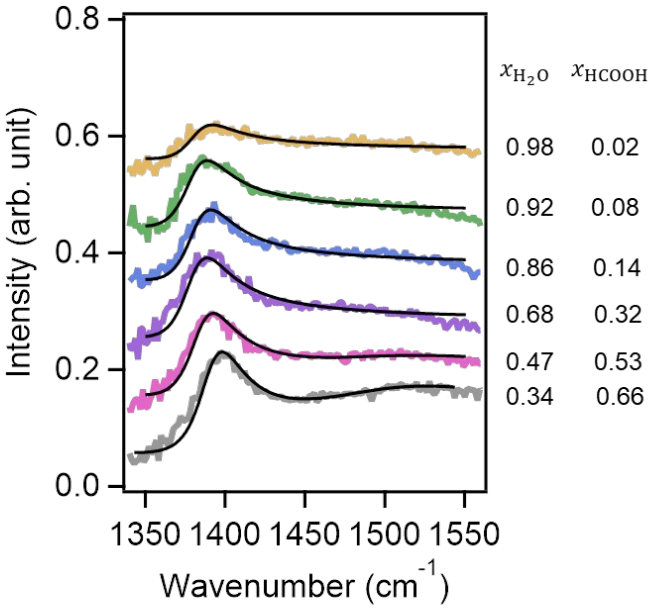

Figure S13. SFG spectra in the H-O-D bending frequency region at the interface of the air-aqueous binary mixture of formic acid. The fits with the Lorentzian model are shown as black solid lines. These data are offset by 0.1 for clarity.

Assuming the C-O-H bending mode contribution of formic acid molecules only appears in high formic acid concentration[28], we fitted the spectra with Lorentzian model as below,

$$\left|\chi^{(2)}_{\mathrm{eff}}(\omega)\right|^2 = \left|\chi^{(2)}_{\mathrm{NR}} + \chi^{(2)}_{\delta(\mathrm{C-H})}(\omega) + \chi^{(2)}_{\delta(\mathrm{H-O-D})}(\omega) + \chi^{(2)}_{\delta(\mathrm{C-O-H})}(\omega)\right|^2 \tag{S7}$$

The phase of the non-resonant contribution (ϕ_{NR}) was constrained to have the same value for fitting all the spectra. The frequencies of the C-O-H and the H-O-D bending modes were set to have the same value between 0.53 and 0.66 mole fraction data of formic acid. The number of H-O-D in isotopically diluted is lower, compared to light water (a 1:1 H_2O: D_2O mixture consists of ~50% of HOD and ~25% of H_2O and D_2O), makes it extremely challenging to

resolve the weak negative contribution of bend 1 for the isotopic mixtures. Therefore, in the current work, we neglect the bend 1 contribution for the isotopic mixtures. The obtained fitting parameters are displayed in Tables S10 and S11.

Table S10. The spectral fitting parameters for the SFG spectra at the interfaces of isotopically diluted water-formic acid mixtures given in Fig. 3c. The values of x_{HCOOH}, $x_{\mathrm{H_2O}}$ and $x_{\mathrm{D_2O}}$ are the concentration before mixing.

x_{HCOOH}	**0.00**	**0.02**	**0.08**	**0.14**	**0.32**	**0.53**	**0.66**
$x_{\mathrm{H_2O}}$	**0.50**	**0.49**	**0.46**	**0.43**	**0.34**	**0.12**	**0.00**
$x_{\mathrm{D_2O}}$	**0.50**	**0.49**	**0.46**	**0.43**	**0.34**	**0.35**	**0.34**
A_{NR}	-0.18	-0.20	-0.18	-0.19	-0.19	-0.19	-0.21
Φ_{NR}(rad)	-0.36	-0.36	-0.36	-0.36	-0.36	-0.36	-0.36
$A_{\delta(\mathrm{C\text{-}H})}$	—	-1.21	-2.36	-2.36	-2.57	-2.57	-2.87
$\omega_{\delta(\mathrm{C\text{-}H})}(\mathrm{cm^{-1}})$	—	1381	1380	1382	1380	1384	1391
$\Gamma_{\delta(\mathrm{C\text{-}H})}(\mathrm{cm^{-1}})$	—	18	18	18	18	18	18
$A_{\delta(\mathrm{H\text{-}O\text{-}D})}$	-4.85	-5.28	-5.49	-5.64	-4.76	-3.74	-2.64
$\omega_{\delta(\mathrm{H\text{-}O\text{-}D})}(\mathrm{cm^{-1}})$	1473	1471	1463	1462	1457	1456	1456
$\Gamma_{\delta(\mathrm{H\text{-}O\text{-}D})}(\mathrm{cm^{-1}})$	57	57	57	57	57	57	57
$A_{\delta(\mathrm{C\text{-}O\text{-}H})}$	—	0.00	0.00	0.00	0.00	-2.68	-5.61
$\omega_{\delta(\mathrm{C\text{-}O\text{-}H})}(\mathrm{cm^{-1}})$	—	1488	1488	1488	1488	1488	1488
$\Gamma_{\delta(\mathrm{C\text{-}O\text{-}H})}(\mathrm{cm^{-1}})$	—	79	79	79	79	79	79

Table S11. The spectral fitting parameters for the SFG spectra at the interfaces of H_2O-formic acid mixtures given in Figure S13.

x_{HCOOH}	0.00	0.02	0.08	0.14	0.32	0.53	0.66
x_{H_2O}	1.00	0.98	0.92	0.86	0.68	0.47	0.34
A_{NR}	—	-0.19	-0.18	-0.20	-0.20	-0.21	-0.22
Φ_{NR}(rad)	—	-0.36	-0.36	-0.36	-0.36	-0.36	-0.36
$A_{\delta(C\text{-}H)}$	—	-1.21	-2.36	-2.36	-2.57	-2.57	-2.87
$\omega_{\delta(C\text{-}H)}$(cm^{-1})	—	1381	1380	1382	1380	1384	1391
$\Gamma_{\delta(C\text{-}H)}$(cm^{-1})	—	18	18	18	18	18	18
$A_{\delta(C\text{-}O\text{-}H)}$	—	0.00	0.00	0.00	0.00	-2.68	-5.61
$\omega_{\delta(C\text{-}O\text{-}H)}$(cm^{-1})	—	1488	1488	1488	1488	1488	1488
$\Gamma_{\delta(C\text{-}O\text{-}H)}$(cm^{-1})	—	79	79	79	79	79	79

9. Estimation of Water Mole Fraction in the Water-Formic Acid Mixture

The equilibria of the isotopically labeled water-formic acid binary mixture can be written as

$$H_2O + D_2O \rightleftharpoons 2HOD \tag{S8}$$

$$HCOOH + \frac{1}{2}D_2O \rightleftharpoons HCOOD + \frac{1}{2}H_2O \tag{S9}$$

The equilibration constant for expression (S8) is known to be $K = 3.86$,[29] while the equilibrium constant for expression (S9) was assumed to be $K = 1$, based on the rule of the geometric mean.[30] The obtained x_{H_2O}, x_{HOD}, x_{D_2O}, x_{HCOOH} and x_{HCOOD} are listed in Table S12.

Table S12. The obtained x_{H_2O}, x_{HOD}, x_{D_2O}, x_{HCOOH} and x_{HCOOD} in isotopically diluted water-formic acid mixtures.

Before mixing			After mixing				
x_{HCOOH}	x_{H_2O}	x_{D_2O}	x_{HCOOH}	x_{HCOOD}	x_{H_2O}	x_{D_2O}	x_{HOD}
0.000	0.500	0.500	0.000	0.000	0.500	0.500	0.500
0.024	0.488	0.488	0.010	0.010	0.252	0.242	0.486
0.078	0.461	0.461	0.042	0.038	0.252	0.213	0.455
0.137	0.435	0.435	0.075	0.065	0.251	0.186	0.424
0.323	0.340	0.340	0.190	0.130	0.242	0.113	0.325
0.527	0.118	0.355	0.278	0.252	0.130	0.108	0.232
0.657	0.000	0.343	0.325	0.335	0.083	0.088	0.168

10. MD Simulation Details

We carried out the molecular dynamics (MD) simulation for the interfaces of the air-aqueous binary mixture. We used the GROMACS software for performing the MD simulations.[31] The equation of motion was integrated with a time step of 2 fs. The bond lengths of all the molecules were fixed through the LINCS algorithm.[32] MD simulation was run in the canonical ensemble, and the temperature of the system was controlled through the canonical sampling through velocity rescaling method.[33] The target temperature was set to 300 K. We ran 10 ns MD simulations for equilibrating the systems and sequentially obtained total 70 ns MD trajectories which were used for analyzing the data.

For the water-methanol mixture, we used two different combinations of the force field models: The OPLS-AA force field model for methanol[34] and TIP4P/2005 model for water[35] as well as the Kirkwood-Buff derived force field model for methanol[36] with the SPC/E model for water.[37] The 26.6 Å × 26.6 Å × 350 Å box contained a total of 2000 molecules. We prepared the solutions with x_{H_2O} of 0.11, 0.49, 0.69, 0.87, 0.93, 0.98, and 1.00. Since the

OPLS-AA force field tends to reproduce the experimental data (see section 9 in Supporting Information), we showed the data using the OPLS-AA force field in the main text. The data of the Kirkwood-Buff derived force field model is given in this Supporting Information.

For the water-ethanol mixture, we used two different combinations of the force field models: The OPLS-AA force field model for ethanol[34] and TIP4P/2005 model for water[35] as well as the Kirkwood-Buff derived force field model for ethanol[38,39] with the SPC/E model for water.[37] We prepared the solutions with x_{H_2O} of 0.15, 0.58, 0.76, 0.91, 0.95, 0.98, and 1.00. Since the OPLS-AA force field tends to reproduce the experimental data, we showed the data using the OPLS-AA force field model in the main text. The data of the Kirkwood-Buff derived force field model is given in this Supporting Information.

For the water-formic acid mixture, we used two different combinations of the force field models: the original and modified OPLS-AA force field model for formic acid[34] and the TIP4P/2005 model for water[35]. We prepared the solutions with x_{H_2O} of 0.27, 0.47, 0.68, 0.86, 0.92, 0.98, and 1.00. Since the atom-site charges of formic acid assigned in the OPLS-AA force field model are known to cause the poor reproducibility of the density of the formic acid,[40] we obtained the electrostatic potential fitting charge for the trans-conformation of formic acid from the *ab initio* calculation. The *Ab initio* calculation employed the B3LYP/aug-cc-pVTZ level of theory for optimizing the molecular structure, while the electrostatic potential fit charges were obtained at the Hartree-Fock/6-31G* level of theory, based on the OPLS-AA development procedure.[41] The *ab initio* calculation was carried out with Gaussian16 code.[42]

Furthermore, we modified the 1-4 electrostatic interaction of the formic acid. It is known that the trans-conformation is favored when a formic acid molecule is in the gas-phase, while the cis-conformation is more favored when a water molecule interacts with formic

acid.[43] Thus, the cis-trans energy barrier would be essential to determine the ratio of the cis- and trans-conformations in the water-formic acid solutions. In fact, the frequency shift of the C-H stretching SFG feature at the water-formic acid interface (see Fig. 2a of the main text, Figure S6 and Table S4) indicates the transition from cis-conformation (2922 cm^{-1}) to the trans-conformation (2933 cm^{-1}) of the formic acid. We scaled the 1-4 electrostatic interaction down to 30%, rather than 50% which is used for the OPLS-AA force field model. With the scaling down of the 1-4 interactions by 70%, the force field model could reproduce the energy difference of ~4.3 between the cis- and trans-conformation.[44] Thus, we scaled the 1-4 electrostatic interaction down by 70%.

Here, we note that the accurate force field model of formic acid was developed by Jedlovszky and Turi,[45] while it was not used in this study, because the model of Jedlovsky and Turi assumed the trans-conformation of formic acid. As is evident from the experiment, the cis-conformation is more favored than the trans-conformation, and thus the fixed trans-conformation geometry of formic acid is not suitable for the water-formic acid mixture. In fact, we observed 70% cis-conformation for the < 20% formic acid aqueous solution, while we observed < 50% cis-conformation for the ~70% formic acid aqueous solution.

11. Calculation of $\langle N\cos\theta \rangle$ for C-H of the Organic Components and Free O-H of Water

We calculated the depth profiles of aqueous binary mixture density and orientations of methyl C-H for interfacial methanol and ethanol, as well as C-H for interfacial formic acid with various concentrations. The orientation angle of C-H was defined by the angle between the surface normal and the vector pointing from methyl C atom to the averaged methyl H atom coordinate for methanol and ethanol. For formic acid, the orientation angle was between

the surface normal and C-H bond direction. We defined the free O-H of water by using the geometry parameters (R, β), where R is the intermolecular O···O distance, and β is the angle of H-O···O[46]. When a pair of water molecules satisfy the condition of O···O distance less than 3.5 Å and H-O···O angle smaller than 50°, an hydrogen bond is formed. If a hydrogen atom does not find a hydrogen bond partner, we defined the O-H group, including this H atom as a free O-H group. The orientation angle of free O-H was formed by the angle between the surface normal and the free O-H bond. We calculated the depth profiles of the interfacial water molecules with the free O-H group. The data shown in Fig. 2b and d (solid lines) in the main text were obtained by integrating these depth profiles and normalized by the maximum value among these concentrations.

12. Depth Profiles of Density and Orientation

We calculated the depth profiles of aqueous binary mixture density and orientations for the interfacial water molecules. The origin point of the profiles is set to the center of mass for the whole system. The depth profiles of $\langle N\cos\theta\rangle$ are given in Figure S14. The data shown in Fig. 3d in the main text are obtained by integrating these depth profiles. To compute the orientation of the interfacial molecules, we defined the interfacial regions. This was given as the FWHM region of the depth profiles of $\langle N\cos\theta\rangle$.

Note that here we cannot exclude the possibility that the variation of the number of water molecules (N) upon the addition of organic molecules affects the $\langle N\cos\theta\rangle$ variation at the air-binary mixture interface. However, the discussion on the variation of N itself may mislead the conclusion, while the number of molecules located in the interfacial region is critically affected by the surface nanoroughness, which differs significantly between the three

binary mixtures considered here (Figure S14 and S15). In fact, arbitrary decomposition of N and $\cos\theta$ terms has resulted in a different interpretation of the binary mixture.[12,47,48] Thus, here, we do not discuss the impact of N on the SFG signal.

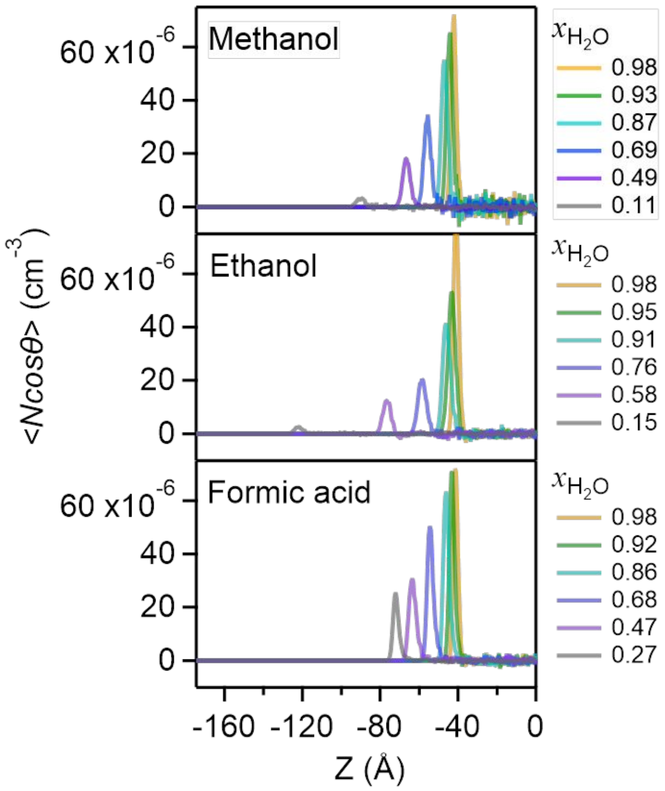

Figure S14. The depth profiles of $\langle N\cos\theta\rangle$ of the interfacial water.

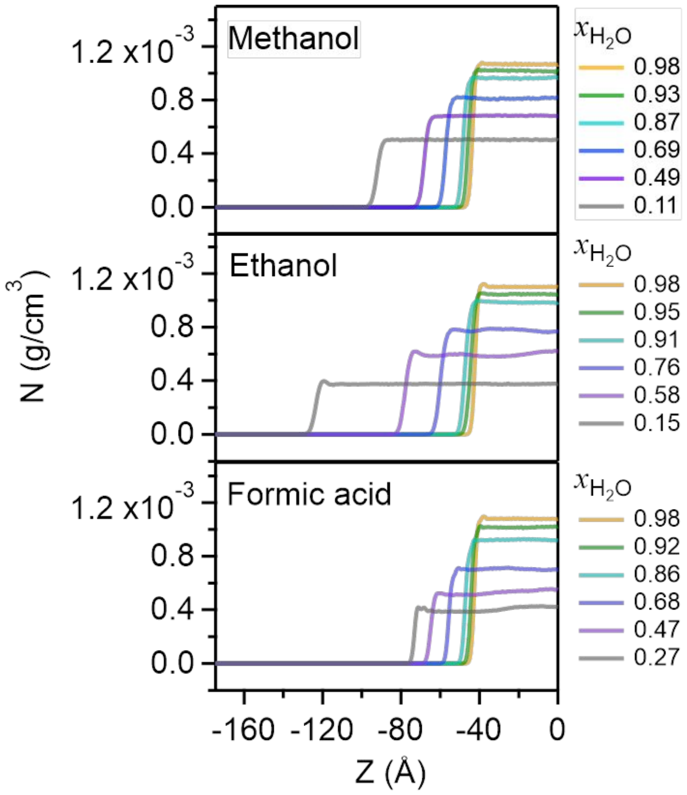

Figure S15. The density profiles of the interfacial water.

13. Comparison of Different Force Field Models

Figure S16 compares the different force field models. For the aqueous binary mixtures of methanol and ethanol, we could not find an obvious difference in the orientation of the interfacial water. On the other hand, it is clear that the original OPLS-AA force field model of formic acid together with the TIP4P/2005 model of water does not reproduce the experimental data. In fact, the OPLS parameterization is known to reproduce the physical properties of formic acid poorly.[40]

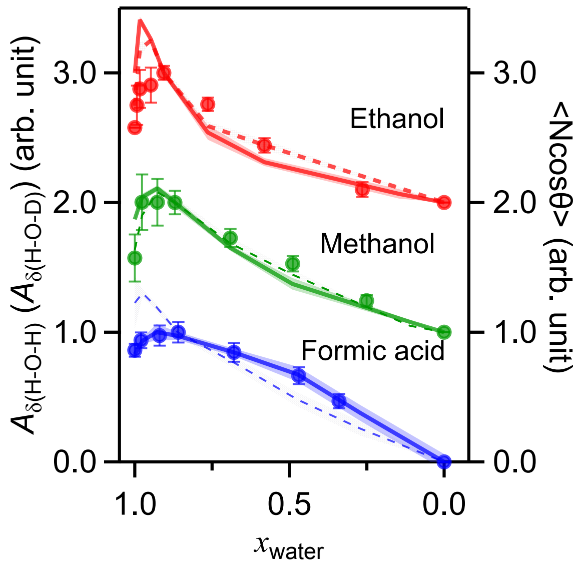

Figure S16. The comparison of different simulation methods for the water's bending mode at the interfaces of air-aqueous binary mixtures. Solid area indicates simulation data from a combination of OPLS-AA for methanol/ethanol and TIP4P/2005 for water and a combination of the modified OPLS-AA for formic acid and TIP4P/2005 for water. Mesh shaded area indicates simulation data from a combination of Kirkwood-Buff derived force field model for methanol/ethanol and SPC/E for water and combination of the original OPLS-AA for formic acid and TIP4P/2005 for water. Traces are offset by 1 for clarity.

References

(1) Sommer, T.; Trejbal, J.; Kopecký, D. Isobaric and Isothermal Vapor-Liquid Equilibria for the Binary System of Water + Formic Acid at 99.41 KPa, 388.15 K, and 398.15 K. *J. Chem. Eng. Data* **2016**, *61*, 3398–3405.

(2) Antoine, M. C. Tensions Des Vapeurs; Nouvelle Relation Entre Les Tensions et Les Températures [Vapor Pressure: A New Relationship between Pressure and Temperature]. *Comptes Rendus des Séances l'Académie des Sci. (in French)* **1888**, *107*, 681–685.

(3) Poling, B. E.; Prausnitz, J. M.; O'Connell, J. P. The Properties of Gases and Liquids. *McGraw-Hill Educ.* **2001**.

(4) Kurihara, K.; Minoura, T.; Takeda, K.; Kojima, K. Isothermal Vapor-Liquid Equilibria for Methanol + Ethanol + Water, Methanol + Water, and Ethanol + Water. *J. Chem. Eng. Data* **1995**, *40*, 679–684.

(5) Nitsche, M.; Gbadamosi, R. Practical Column Design Guide. *Springer* **2017**.

(6) Lu, Z.; Kinefuchi, I.; Wilke, K. L.; Vaartstra, G.; Wang, E. N. A Unified Relationship for Evaporation Kinetics at Low Mach Numbers. *Nat. Commun.* **2019**, *10*, 2368.

(7) Milev, A. S.; Wilson, M. A.; Kannangara, G. S. K.; Feng, H.; Newman, P. A. Isothermal Evaporation of Ethanol in a Dynamic Gas Atmosphere. *J. Phys. Chem. A* **2012**, *116*, 150–157.

(8) Ohto, T.; Backus, E. H. G.; Mizukami, W.; Hunger, J.; Bonn, M.; Nagata, Y. Unveiling the Amphiphilic Nature of TMAO by Vibrational Sum Frequency Generation Spectroscopy. *J. Phys. Chem. C* **2016**, *120*, 17435–17443.

(9) Ohto, T.; Hunger, J.; Backus, E. H. G.; Mizukami, W.; Bonn, M.; Nagata, Y. Trimethylamine-N-Oxide: Its Hydration Structure, Surface Activity, and Biological Function, Viewed by Vibrational Spectroscopy and Molecular Dynamics Simulations. *Phys. Chem. Chem. Phys.* **2017**, *19*, 6909–6920.

(10) Wang, C.; Groenzin, H.; Shultz, M. J. Surface Characterization of Nanoscale TiO_2 Film by Sum Frequency Generation Using Methanol as a Molecular Probe. *J. Phys. Chem. B* **2004**, *108*, 265–272.

(11) Lu, R.; Gan, W.; Wu, B. H.; Zhang, Z.; Guo, Y.; Wang, H. F. C-H Stretching Vibrations of Methyl, Methylene and Methine Groups at the Vapor/Alcohol (n = 1-8) Interfaces. *J. Phys. Chem. B* **2005**, *109*, 14118–14129.

(12) Li, X.; Liu, J.; Lin, K.; Zhang, Y.; Zhang, Y.; Zheng, R.; Shi, Q.; Guo, Y.; Lu, Z. Toward Unraveling the Puzzle of Sum Frequency Generation Spectra at Interface of Aqueous Methanol Solution: Effects of Concentration-Dependent Hyperpolarizability. *J. Phys. Chem. C* **2019**, *123*, 12975–12983.

(13) Ma, G.; Allen, H. C. Surface Studies of Aqueous Methanol Solutions by Vibrational Broad Bandwidth Sum Frequency Generation Spectroscopy. *J. Phys. Chem. B* **2003**, *107*, 6343–6349.

(14) Liu, J.; Li, X.; Hou, J.; Li, X.; Lu, Z. The Influence of Sodium Iodide Salt on the Interfacial Properties of Aqueous Methanol Solution by a Combined Molecular Simulation and Sum Frequency Generation Vibrational Spectroscopy Study. *Langmuir* **2019**, *35*, 7050–7059.

(15) Sun, S.; Tang, F.; Imoto, S.; Moberg, D. R.; Ohto, T.; Paesani, F.; Bonn, M.; Backus, E. H. G.; Nagata, Y. Orientational Distribution of Free O-H Groups of Interfacial

Water Is Exponential. *Phys. Rev. Lett.* **2018**, *121*, 246101.

(16) Hou, J.; Zhang, X.; Lu, Z. Comparing Vibrational Sum Frequency Generation Responses at Fused Silica and Fluorite/Liquid Ethanol Interfaces. *Chem. Phys.* **2020**, *536*, 110814.

(17) Johnson, C. M.; Tyrode, E.; Kumpulainen, A.; Leygraf, C. Vibrational Sum Frequency Spectroscopy Study of the Liquid/Vapor Interface of Formic Acid/Water Solutions. *J. Phys. Chem. Lett.* **2009**, *113*, 13209–13218.

(18) Kananenka, A. A.; Skinner, J. L. Fermi Resonance in OH-Stretch Vibrational Spectroscopy of Liquid Water and the Water Hexamer. *J. Chem. Phys.* **2018**, *148*, 244107.

(19) De Marco, L.; Ramasesha, K.; Tokmakoff, A. Experimental Evidence of Fermi Resonances in Isotopically Dilute Water from Ultrafast Broadband IR Spectroscopy. *J. Phys. Chem. B* **2013**, *117*, 15319–15327.

(20) Schaefer, J.; Backus, E. H. G.; Nagata, Y.; Bonn, M. Both Inter- and Intramolecular Coupling of O−H Groups Determine the Vibrational Response of the Water/Air Interface. *J. Phys. Chem. Lett* **2016**, *7*, 4591–4595.

(21) Ni, Y.; Skinner, J. L. IR and SFG Vibrational Spectroscopy of the Water Bend in the Bulk Liquid and at the Liquid-Vapor Interface, Respectively. *J. Chem. Phys.* **2015**, *143*, 014502.

(22) Nagata, Y.; Hsieh, C.-S.; Hasegawa, T.; Voll, J.; Backus, E. H. G.; Bonn, M. Water Bending Mode at the Water–Vapor Interface Probed by Sum-Frequency Generation Spectroscopy: A Combined Molecular Dynamics Simulation and Experimental Study.

J. Phys. Chem. Lett. **2013**, *4*, 1872–1877.

(23) Moberg, D. R.; Straight, S. C.; Paesani, F. Temperature Dependence of the Air/Water Interface Revealed by Polarization Sensitive Sum-Frequency Generation Spectroscopy. *J. Phys. Chem. B* **2018**, *122*, 4356–4365.

(24) Dutta, C.; Benderskii, A. V. On the Assignment of the Vibrational Spectrum of the Water Bend at the Air/Water Interface. *J. Phys. Chem. Lett.* **2017**, *8*, 801–804.

(25) Seki, T.; Sun, S.; Zhong, K.; Yu, C. C.; Machel, K.; Dreier, L. B.; Backus, E. H. G.; Bonn, M.; Nagata, Y. Unveiling Heterogeneity of Interfacial Water through the Water Bending Mode. *J. Phys. Chem. Lett.* **2019**, *10*, 6936–6941.

(26) Millikan, R. C.; Pitzer, K. S.; Millikan, B. R. C.; Pitzer, K. S.; Millikan, R. C. The Infrared Spectra of Dimeric and Crystalline Formic Acid. *J. Am. Chem. Soc.* **1958**, *80*, 3515–3521.

(27) Gadermann, M.; Vollmar, D.; Signorell, R. Infrared Spectroscopy of Acetic Acid and Formic Acid Aerosols: Pure and Compound Acid/Ice Particles. *Phys. Chem. Chem. Phys* **2007**, *9*, 4535–4544.

(28) Lu, Z.; Karakoti, A.; Velarde, L.; Wang, W.; Yang, P.; Thevuthasan, S.; Wang, H. F. Dissociative Binding of Carboxylic Acid Ligand on Nanoceria Surface in Aqueous Solution: A Joint in Situ Spectroscopic Characterization and First-Principles Study. *J. Phys. Chem. C* **2013**, *117*, 24329–24338.

(29) Meija, J.; Mester, Z.; D'Ulivo, A. Mass Spectrometric Separation and Quantitation of Overlapping Isotopologues. H2O/HOD/D2O and H2Se/HDSe/D2Se Mixtures. *J. Am. Soc. Mass Spectrom.* **2006**, *17*, 1028–1036.

(30) Bigeleisen, J. Statistical Mechanics of Isotopic Systems with Small Quantum Corrections. I. General Considerations and the Rule of the Geometric Mean. *J. Chem. Phys.* **1955**, *23*, 2264–2267.

(31) Abraham, M. J.; Murtola, T.; Schulz, R.; P´all, S.; Smith, J. C.; Hess, B.; Lindahl, E. GROMACS : High Performance Molecular Simulations through Multi-Level Parallelism from Laptops to Supercomputers. *SoftwareX* **2015**, *2*, 19–25.

(32) Hess, B.; Bekker, H.; Berendsen, H. J. C.; Fraaije, J. G. E. M. LINCS : A Linear Constraint Solver for Molecular Simulations. *J. Comput. Chem.* **1997**, *18*, 1463–1472.

(33) Bussi, G.; Donadio, D.; Parrinello, M. Canonical Sampling through Velocity Rescaling. *J. Chem. Phys.* **2007**, *126*, 014101.

(34) Jorgensen, W. L.; Maxwell, D. S.; Tirado-Rives, J. Development and Testing of the OPLS All-Atom Force Field on Conformational Energetics and Properties of Organic Liquids. *J. Am. Chem. Soc.* **1996**, *118*, 11225–11236.

(35) Abascal, J. L. F.; Vega, C. A General Purpose Model for the Condensed Phases of Water: TIP4P/2005. *J. Chem. Phys.* **2005**, *123*, 234505.

(36) Weerasinghe, S.; Smith, P. E. A Kirkwood-Buff Derived Force Field for Methanol and Aqueous Methanol Solutions. *J. Phys. Chem. B* **2005**, *109*, 15080–15086.

(37) Berendsen, H. J. C.; Grigera, J. R.; Straatsma, T. P. The Missing Term in Effective Pair Potentials. *J. Phys. Chem.* **1987**, *91*, 6269–6271.

(38) Mijaković, M.; Polok, K. D.; Kežić, B.; Sokolić, F.; Perera, A.; Zoranić, L. A Comparison of Force Fields for Ethanol-Water Mixtures. *Mol. Simul.* **2015**, *41*, 699–712.

(39) Jiao, Y. The Development of Accurate Force Fields for Protein Simulation [PhD Thesis], Manhattan (KS): Kansas State University, 2012.

(40) Caleman, C.; Maaren, P. J. Van; Hong, M.; Hub, J. S.; Costa, L. T.; Spoel, D. Van Der. Force Field Benchmark of Organic Liquids: Density, Enthalpy of Vaporization, Heat Capacities, Surface Tension, Isothermal Compressibility, Volumetric Expansion Coefficient, and Dielectric Constant. *J. Chem. Theory Comput.* **2012**, *8*, 61–74.

(41) Watkins, E. K.; Jorgensen, W. L. Perfluoroalkanes: Conformational Analysis and Liquid-State Properties from Ab Initio and Monte Carlo Calculations. *J. Phys. Chem. A* **2001**, *105*, 4118–4125.

(42) Frisch, M. J.; Trucks, G. W.; Schlegel, H. E.; Scuseria, G. E.; Robb, M. A.; Cheeseman, J. R.; Scalmani, G.; Barone, V.; Petersson, G. A.; O., F.; et al. Gaussian 16. *Gaussian, Inc., Wallingford CT,*. 2016.

(43) Marushkevich, K.; Khriachtchev, L.; Räsänen, M. Hydrogen Bonding between Formic Acid and Water: Complete Stabilization of the Intrinsically Unstable Conformer. *J. Phys. Chem. A* **2007**, *111*, 2040–2042.

(44) Hirao, H. Theoretical Study of Formic Acid: A New Look at the Origin of the Planar Z Conformation and C-O Rotational Barrier. *Chem. Phys.* **2008**, *344*, 213–220.

(45) Jedlovszky, P.; Turi, L. A New Five-Site Pair Potential for Formic Acid in Liquid Simulations. *J. Phys. Chem. A* **1997**, *101*, 2662–2665.

(46) Tang, F.; Ohto, T.; Hasegawa, T.; Xie, W. J.; Xu, L.; Bonn, M.; Nagata, Y. Definition of Free O-H Groups of Water at the Air-Water Interface. *J. Chem. Theory Comput.* **2018**, *14*, 357–364.

(47) Ishihara, T.; Ishiyama, T.; Morita, A. Surface Structure of Methanol/Water Solutions via Sum Frequency Orientational Analysis and Molecular Dynamics Simulation. *J. Phys. Chem. C* **2015**, *119*, 9879–9889.

(48) Ishiyama, T.; Takagi, S.; Hirano, T.; Wang, L.; Morita, A. Comment on "Toward Unraveling the Puzzle of Sum Frequency Effects of Concentration-Dependent Hyperpolarizability." *J. Phys. Chem. C* **2020**, *123*, 12975−12983.
